# Miniaturized Analytical Strategy Based on μ-SPEed for Monitoring the Occurrence of Pyrrolizidine and Tropane Alkaloids in Honey

**DOI:** 10.1021/acs.jafc.3c04805

**Published:** 2023-12-18

**Authors:** Natalia Casado, Sonia Morante-Zarcero, Isabel Sierra

**Affiliations:** † Departamento de Tecnología Química y Ambiental, E.S.C.E.T, Universidad Rey Juan Carlos, C/Tulipán s/n, 28933 Móstoles, Madrid, Spain; ‡ Instituto de Tecnologías para la Sostenibilidad, Universidad Rey Juan Carlos, C/Tulipán s/n, 28933 Móstoles, Madrid, Spain

**Keywords:** natural toxins, alkaloids, green analytical chemistry, microextraction, UHPLC–IT-MS/MS, food safety

## Abstract

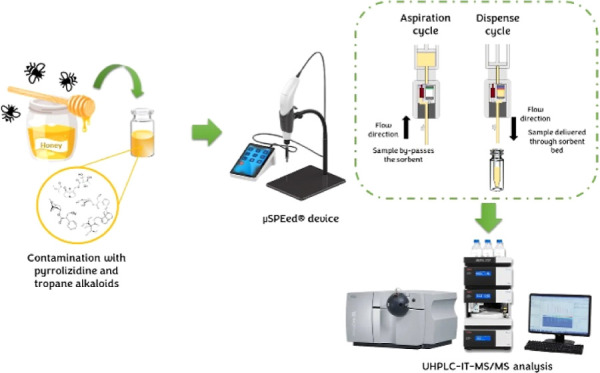

Currently, the analysis of trace-level contaminants in food must be addressed following green analytical chemistry principles and with a commitment to the sustainable development goals. Accordingly, a sustainable and ecofriendly microextraction procedure based on μ-SPEed followed by ultrahigh liquid chromatography coupled to ion-trap tandem mass spectrometry analysis was developed to determine the occurrence of pyrrolizidine and tropane alkaloids in honey samples. The μ-SPEed procedure took approximately 3 min per sample, using only 100 μL of organic solvent and 300 μL of diluted sample. The method was properly validated (overall recoveries 72–100% and precision RSD values ≤15%), and its greenness was scored at 0.61 out of 1. The method was applied to different honey samples, showing overall contamination levels from 32 to 177 μg/kg of these alkaloids. Atropine was found in all the samples, whereas retrorsine *N*-oxide, lasiocarpine, echimidine, and echimidine *N*-oxide were the main pyrrolizidine alkaloids in the samples analyzed.

## 1 Introduction

Food safety is one of the main public health concerns worldwide, which is placed at the top of the priorities of the World Health Organization (WHO) and is included as part of the sustainable development goal (SDG) no. 2 of the 2030 Agenda.^1,2^ For these reasons, special attention should be paid to the presence of trace-level contaminants in food due to the short/long-term risks that their intake may entail for the health of consumers.^3,4^ Contaminants that may be present in food can be chemical or biological. Among chemical contaminants, natural toxins have aroused increasing interest in recent years because of their potential hazards for human health related to their underestimated occurrence in different foodstuffs.^5^ Within these natural toxins, the pyrrolizidine alkaloids (PAs) and the tropane alkaloids (TAs) can be especially highlighted as many food alerts have notified the presence of these compounds at concentration levels higher than the maximum levels set by the competent authorities in food.^6^ These alkaloids are basic compounds synthesized as secondary metabolites from different plant species. They can be extremely dangerous if ingested without control, leading to the possible appearance of acute intoxications or chronic diseases.^7^ In consequence, awareness has been raised by the European Food Safety Authority,^8−15^ and maximum concentration limits for these alkaloids have been regulated in some food products to keep the exposure of consumers to these toxins as low as possible through the diet.^16^ Accordingly, there is a need for sensitive, selective, fast, affordable, and effective analytical approaches for the evaluation of these alkaloids that threaten food safety.

Considering that similar contamination pathways have been described for PAs and TAs, it seems suitable to develop analytical strategies that enable the simultaneous determination of these two types of alkaloids, since they may appear as contaminants in the same products.^6^ In this context, honey can be highlighted among the different products where PAs and TAs can be found as food contaminants.^17,18^ Their occurrence is due to pollen dislodged into nectar by bees during the pollination process. Accordingly, several authors have evaluated the occurrence of PAs and TAs in honey samples.^19−38^ However, all of these works performed conventional sample preparation techniques, such as solid-phase extraction (SPE) or QuEChERS, leading to relatively high volumes of organic solvents, the use of different types of reagents that increase the cost and the waste residues, and the need for multiple steps, which are usually time-consuming. In this context, in the food analysis field, there is currently a demand to improve sample preparation methods, replacing traditional ones for other approaches with better advantages, such as fewer time requirements, higher extraction efficiency, a lower level of chemical consumption, good affordability, and cost-effectiveness, among others.^4^ Moreover, considering the worldwide concern to prevent environmental pollution, which has reached serious dimensions globally, there is a current trend toward green chemistry to develop more sustainable processes,^39^ which also involves a commitment with other SDGs, such as SDG no. 12 (ensure sustainable consumption and production patterns) and SDG no. 13 (take urgent action to combat climate change and its impacts).^2^ Hence, the miniaturization of the sample preparation techniques has emerged as an interesting and green chemistry alternative to conventional methods.^40^

Miniaturized approaches, normally called microextraction methods, involve the reduction of system dimensions without losing efficiency. Among the different microextraction procedures derived from traditional SPE, the μ-SPEed technique developed by the EPREP company (Victoria, Australia) stands out significantly. The μ-SPEed is an adsorption procedure very similar to the microextraction by packed sorbents, but with important improvements.^41^ In the μ-SPEed, small sorbent particles (<3 μm) are used, which provide a high surface area that improves the interaction with the analytes, leading to high extraction efficiency. This sorbent is tightly packed in a microcartridge equipped with a needle and a one-way pressure-driven valve that dispenses the sample flow in a single direction at high pressure (up to 1600 psi). Thanks to this valve, the sample is aspirated through the cartridge needle into the syringe barrel, and then under well controlled flow conditions, the sample is pushed at high pressure through the sorbent bed. Therefore, the μ-SPEed cartridges act like a short HPLC column, where the flow always comes from the top of the sorbent bed. Thus, μ-SPEed could be considered a high-resolution miniaturized SPE, providing more efficiency and faster procedures at a lower overall cost than conventional SPE processes. This overall reduced cost is mainly referred to the lower amount of solvents and sorbents needed during the extraction, which increases the cost-effectiveness of the procedure. Nonetheless, to perform μ-SPEed, apart from the cartridges, an initial investment in the digital device may be required (similar price to that of medium laboratory equipment). However, conventional SPE also needs the investment of a vacuum manifold and a pump besides the SPE cartridges. Moreover, it is also possible to perform μ-SPEed in a manual way only using the μ-SPEed cartridge and a manual syringe. On the other hand, μ-SPEed also enables to achieve good preconcentration of the analytes, delivering the sample extract ready for direct chromatographic or mass spectrometry injection, without requiring and evaporation-reconstitution step prior to instrumental analysis as usually in SPE. To date, this technique has been successfully applied in the food analysis field for the extraction of different compounds from different food matrices.^42−48^

To the best of our knowledge, only the work of Celano et al. has proposed a miniaturized method based on dispersive liquid–liquid microextraction (DLLME) for the determination of PAs in honey samples,^22^ but to date, there are no published works that propose the miniaturized extraction and determination of both PAs and TAs jointly. Hence, this work proposes the development of a sustainable and green microextraction methodology based on μ-SPEed followed by ultra-high-performance liquid chromatography coupled to ion-trap tandem mass spectrometry (UHPLC–IT-MS/MS) analysis to determine the simultaneous occurrence of 21 PAs and 2 TAs in honey samples. This strategy aims to be an advancement and improvement over the current conventional sample preparation methods available in the literature, providing important benefits that minimize the environmental impact as well as simplicity and quickness in its operation to simultaneously monitor the presence of these two types of alkaloids in food samples.

## 2 Materials and Methods

### 2.1 Chemicals, Materials, and Standard Solutions

Methanol (MeOH) and acetonitrile (ACN) for LC–MS, sulfuric acid 98%, and dimethyl sulfoxide (DMSO) were purchased from Scharlab (Barcelona, Spain). Ammonia solution 25% for LC–MS was acquired from Merck KGaA (Darmstadt, Germany). Formic acid for LC–MS was obtained from Fluka (Busch, Switzerland). A Millipore Milli-Q System (Billerica, MA, USA) was used to obtain ultrapure Milli-Q water (resistivity of 18.2 MΩ cm). For μ-SPEed, an electronic digiVol digital syringe (500 μL) acquired from EPREP (Mulgrave, Victoria, Australia) was used. μ-SPEed cartridges: octadecylsilane (C18 silica-based, 4 mg, 3 μm, 120 Å) and porous cross-linked polystyrene divinylbenzene (PS/DVB, 4 mg, 3 μm, 300 Å) were also obtained from EPREP (Mulgrave, Victoria, Australia).

The standards of PAs and TAs used were of high purity grade (≥90%). Retrorsine, scopolamine hydrobromide, and atropine sulfate were acquired from Sigma-Aldrich (St. Louis, MO, USA). The rest of PAs and their related *N*-oxides (PANOs) were obtained from PhytoLab GmbH & Co. KG (Vestenbergsgreuth, Germany). Individual standard solutions (1000 mg/L) were prepared by diluting suitable amounts of each compound in an appropriate solvent according to their solubility. Thus, intermedine *N*-oxide, lycopsamine *N*-oxide, echimidine, echimidine *N*-oxide, lasiocarpine, lasiocarpine *N*-oxide, retrorsine *N*-oxide, senecionine *N*-oxide, senecivernine, senecivernine *N*-oxide, seneciphylline *N*-oxide, senkirkine, and the two TAs were prepared in MeOH. Intermedine, lycopsamine, europine, europine *N*-oxide, retrorsine, heliotrine, heliotrine *N*-oxide, senecionine, and seneciphylline were diluted in DMSO/ACN (1/4, v/v). From the individual stock standard solutions, a mix-standard working solution containing all the PAs, PANOs, and TAs was prepared at the desired concentration in MeOH. All the standard solutions were stored at −20 °C in the dark.

### 2.2 Honey Samples

Commercial and retail honey samples of different types (monofloral and multifloral) were analyzed in the present work. Five commercial honey samples (including 3 rosemary honeys, 1 orange blossom honey, and 1 multifloral honey) were purchased at different stores from Spain and Israel. Two retail honey samples (including 1 multifloral honey and 1 woodland honey) were acquired in a town in western Spain (Extremadura). The details of samples (code name, geographical, and botanical origin) are listed in Table S1.

### 2.3 Sample Preparation

All of the samples were homogenized by manual stirring with a spatula. Representative 0.5 g of each sample was weighed into a 10 mL vial and dissolved in 2.5 mL of 0.05 M sulfuric acid. The samples were magnetically stirred for 15 min to achieve complete dissolution. Afterward, they were filtered through a 0.45 μm PTFE filter membrane and stored at 4 °C until microextraction.

Under the final conditions, the microextraction of the honey sample extracts was performed by using the μ-SPEed digital syringe coupled to a PS/DVB sorbent cartridge. First, the sorbent cartridge was conditioned with two aspiration-dispense cycles of 100 μL of water, followed by two aspiration-dispense cycles of 100 μL of 0.05 M sulfuric acid aqueous solution. In the conditioning step, the aspiration flow rate was automatically established at 20 μL/s, whereas the dispense flow rate was set to 10 μL/s to avoid overpressure problems. Afterward, sample loading was carried out in extract-discard operation mode using three aspiration-dispense cycles of 100 μL of the honey sample extract (300 μL in total). In this step, the aspiration-dispense flow rate was automatically set at 10 μL/s to avoid cavitation and overpressure, as well as to ensure proper interaction between the analytes and the sorbent. No washing step was performed to avoid the loss of the analytes. Hence, after the loading step, the analytes were directly eluted from the sorbent into a chromatographic vial with 100 μL of MeOH (preconcentration factor of 3). The aspiration-dispense flow rate in the elution step was automatically set at 10 μL/s. To avoid memory effects (carry-over), after each extraction, the cartridge was rinsed with 2 × 250 μL of MeOH using an aspiration-dispense flow rate automatically set at 20 μL/s. Each honey sample was extracted and analyzed in triplicate.

### 2.4 UHPLC–MS/MS Analysis

The chromatographic vials containing the honey sample extracts were analyzed on a UHPLC system (Dionex UltiMate 3000, Thermo Scientific, Waltham, MA, USA) coupled to an ion-trap tandem mass spectrometer (ESI-ITMS amaZon SL, Bruker, Billerica, MA, USA). The chromatographic separation was performed on a Luna Omega Polar C18 column (100 mm × 2.1 mm, 1.6 μm particle size, Phenomenex, Torrance, CA, USA) at 30 °C. The mobile phase consisted of water containing 0.2% formic acid (solvent A) and MeOH containing 0.2% ammonia (solvent B). The separation was achieved at a flow rate of 0.3 mL/min with the following elution gradient: 0–0.5 min 5% B, 0.5–3 min 10% B, 3–7 min 25% B, 7–9 min 30% B, 9–12 min 70% B, and 12–14 min 5% B, and held for 1 min for re-equilibration to initial conditions. The total analysis time was 15 min, and 5 μL was used as injection volume. Under these chromatographic conditions, the separation of the 21 PAs/PANOs and 2 TAs was achieved in less than 14 min (Table 1).

**Table 1:** Retention Time, Mass Spectrum Parameters, Precursor Ion, and Product Ions of the Target Analytes

analyte	retention time (min)	precursor ion (*m*/*z*)	isolation (width)	reaction cut-off	fragmentation amplitude	fragments (MS^2^, product ions (*m*/*z*))^a^
intermedine	6.3	300	4.0	90	0.70	**137.63**/155.5/119.75/93.88/209.5/255.38/237.38/281.5
lycopsamine	6.7	300	4.0	90	0.70	**137.63**/155.63/119.75/94.00/209.5/255.38/237.38/281.38
europine	6.8	330	4.0	120	0.80	**253.38**/137.63/155.63/311.38/239.38/106.25/235.38
europine *N*-oxide	7.5	346	4.0	120	0.80	**327.38**/171.5/269.38/255.38/287.38/241.38/135.63/136.63/137.63/154.63/153.63/211.5/227.38/295.38/227.5/251.38
scopolamine	7.6	304	4.0	90	1.00	**137.75**/155.63/109.75/240.88/273.75
intermedine *N*-oxide	7.8	316	4.0	100	0.80	**171.5**/225.38/137.63/135.63/136.63/271.38/154.5/153.5/155.5/297.38/209.5/199.38/253.38/110.75/119.75
lycopsamine *N*-oxide	8.1	316	4.0	100	0.80	**171.5**/225.38/137.63/135.63/136.63/271.38/154.5/153.5/155.5/297.38/209.5/199.38/253.38/110.75/119.75
retrorsine	8.7	352	4.0	120	0.80	**323.38**/275.38/137.63/305.38/303.38/219.50/150.5/168.5
retrorsine *N*-oxide	9.0	368	4.0	130	0.90	**245.38**/217.5/339.38/219.5/201.5/291.38/135.63/177.5/173.5/319.38/151.63/153.63/269.38/349.38/337.38/191.5/227.38/333.38/275.38/305.38/309.38
seneciphylline	9.3	334	4.0	110	0.80	**305.38**/119.75/287.38/137.63/150.5/245.38/273.38/117.75/121.75/171.63
heliotrine	9.4	314	4.0	100	0.70	**137.63**/119.75/155.63/151.63/281.38/253.25
seneciphylline *N*-oxide	9.9	350	4.0	100	0.80	**322**/117.75/245.88/135.75/332/287.88/153.75/166.63/177.75/217.75/201.75/227.75/105.88
heliotrine *N*-oxide	10.1	330	4.0	120	1.00	**171.5**/135.63/136.66/137.68/153.63/154.63/123.75/297.38
atropine	10.2	290	4.0	85	0.95	**123.75**/259.75/92.88/90.88/213.75/241.75
senecivernine	10.6	336	4.0	110	0.80	**307.38**/119.75/137.63/289.38/152.63/219.38/237.38/247.38/117.75/121.75
senecionine	10.8	336	4.0	110	0.80	**307.38**/119.75/137.63/289.38/152.63/219.38/237.38/247.38/117.75/121.75
senecivernine *N*-oxide	11.1	352	4.0	120	0.80	**219.75**/324/117.75/245.75/253.88/135.75/105.88/201.75/289.88/153.75/164.63/177.75/191.75/334
senecionine *N*-oxide	11.5	352	4.0	100	1.00	**219.75**/324/117.75/245.75/253.88/135.75/105.88/201.75/289.88/153.75/164.63/177.75/191.75/334
echimidine	12.3	398	4.0	100	0.60	**119.75**/219.75/336/380
echimidine *N*-oxide	12.3	414	4.0	110	0.70	**396**/352/253.38/337.38/335.38
senkirkine	12.6	366	4.0	130	0.80	**167.5**/152.63/149.63/219.5/249.38/267.38/303.38/319.38/337.38/347.38/185.5/136.63/134.63/137.63/139.63/180.5
lasiocarpine	13.2	412	4.0	100	0.70	**335.38**/393.38/219.5/119.75/237.5
lasiocarpine *N*-oxide	13.6	428	4.0	100	0.80	**410**/352/253.88/338/370/370/135.75/119.75/105.88/150.75

a In bold product ion used for quantification, underlined the mandatory product ion used for confirmation.

Ionization was achieved with an electrospray ionization interface (ESI) operating in positive ion mode and with the following settings: capillary voltage of 4500 V, end plate offset of 500 V, nebulizer gas of 20 psi, dry gas flow rate of 10 L/min, and dry temperature of 200 °C. Mass spectra were collected with a mass range 70–700 *m*/*z*, and multiple reaction monitoring scan mode was used for all the target analytes. The mass spectrum parameters of each analyte were individually optimized by direct infusion of each analyte into pure standard solutions (5 μg/mL) at a flow rate of 4 μL/min in the ESI source. Accordingly, the precursor ion of each analyte ([M + H]^+^) was identified, and was then isolated and fragmented to obtain the mass spectrum (MS^2^) with the product ions of each analyte (Table 1). For each analyte, the most intense product ion of the MS^2^ spectrum was selected for quantification, whereas the others (at least one of them was mandatory) were used for confirmation (Table 1).

### 2.5 Validation of the Analytical Method

Analytical parameters of the proposed method, such as selectivity, linearity, method detection limits (MDLs), method detection quantification limits (MQLs), matrix effects (MEs), accuracy, and precision, were validated using the criteria established in the European Commission SANTE/12682/2019 document, regulation EC no. 401/2006, and in the Q2(R1) ICH guidelines.^49−51^ Since no honey samples completely free of PAs and TAs were found, the validation assays were performed using sample CR_1.

Selectivity was assessed by comparing the spectra of the different analytes obtained from injecting standard solutions to the spectra obtained when injecting the samples. According to the European Commission SANTE/12682/2019 document,^49^ selectivity can be considered satisfactory when the deviation observed in the spectra is less than ±30% and the retention time of the analytes is within the interval ±2.5%. For linearity, matrix-matched calibration curves were used to correct ME. These curves were prepared at 8 known concentration levels from 0.6 to 300 μg/L (corresponding to the range 1–500 μg/kg expressed as p/p) in three consecutive days and were constructed by linear regression plotting the peak area of the quantification product ion versus the spiked analyte concentration. For their preparation, different aliquots of a working standard solution were added to the sample extracts obtained after the μ-SPEed procedure, according to the concentration level of the calibration curve. As previously mentioned, no honey sample free of PAs and TAs were found, so a nonspiked sample extract (denoted as a blank sample) was also analyzed in a parallel way so that the signals of those analytes that naturally occur in the honey sample could be subtracted. This correction was made to estimate the method limits and for the quantification of the analytes. According to the validation guidelines, a good linearity criterion implies coefficient of determination (*R*^2^) values close to 1.^49,50^ Likewise, to assess ME solvent-based calibration curves were prepared using standard solution at the same 8 calibration levels as in the matrix-matched calibration curves (linear range 0.6–300 μg/L) but without the honey matrix. Accordingly, the ME was calculated as follows: [(slope of matrix – matched curve/slope of solvent – based curve) – 1] × 100. Positive ME values mean a signal increase, while negative values indicate signal suppression. ME values within the range ±20% indicate that analytes could be quantified with solvent-based calibration curves instead of matrix-matched calibration curves. In contrast, ME values out of this range indicate that the matrix error should be considered in the quantification. Likewise, a soft ME is considered when values range between −50% < MEs < −20% and 50% > MEs > 20%, whereas a strong ME is considered when values are below −50% or above 50%.

The method sensitivity was determined through estimation of the MDLs and MQLs. These limits were estimated based on the standard deviation of the response and the slope obtained in the matrix-matched calibration curves for the lowest calibration level. Accordingly, MDL = 3.3 × standard deviation of the response at the lowest calibration level/slope of the calibration curve, and MQL = 10 × standard deviation of the response at the lowest calibration level/slope of the calibration curve.^51^ In those analytes that naturally occur in the samples, before constructing the matrix-matched calibration curves, the response obtained was corrected by subtracting the signal obtained from the blank samples previously mentioned.

For the accuracy and precision of the method, three validation levels (high, medium, and low) were established, corresponding to 500, 50, and 5 μg/kg, respectively. Currently, the maximum limits of PAs and TAs in honey have not yet been set. Hence, these levels were selected based on the maximum levels legislated for these alkaloids in different food products^16^ and on the concentrations found in the literature by previous works (Table S2). Except for liquid products and those intended for infants, the lowest concentration level legislated for TAs is 5 μg/kg, whereas the maximum levels regulated for PAs are higher.^16^ Therefore, 5 μg/kg was chosen as the lowest validation level, while concentrations 10 and 100 times higher were considered for the medium and high levels, respectively. Accordingly, the accuracy was evaluated in terms of recovery at these three validation levels. The recovery was determined by comparing the analytical results of the extracted target analytes from spiked honey samples with the results of simulated samples (nonspiked honey samples subjected to the microextraction procedure and spiked afterward with standards at the same concentration before the chromatographic analysis). The results were expressed as the mean recovery obtained from the analysis of 9 replicates (*n* = 9) extracted on different days. Based on the validation guidelines, for good accuracy, the recovery values must range between 70 and 120%.^49,50^ On the other hand, precision was assessed at the three validation levels in terms of intra-day (repeatability) and inter-day (reproducibility) precision, expressed as relative standard deviation percentage (RSD %). For each validation level, the repeatability was determined by the analysis of 6 replicates (*n* = 6) on the same day, while reproducibility was evaluated by analyzing 3 replicates for 3 consecutive days (*n* = 9). For correct validation, RSD values for method precision must be ≤20%.^49,50^

### 2.6 Confirmation Criteria and Quantification of Analytes in Honey Samples

To confirm the occurrence of the target analytes in the honey samples analyzed, the criteria set in the European Commission SANTE/12682/2019 document was followed.^49^ Accordingly, the results obtained in nonspiked samples should be compared with the results obtained in samples spiked with standard solutions, and confirmation could be determined when: retention time of analytes do not differ more than 0.1 min, product ions selected for confirmation should be detected and must match, and the relative intensity between the product ions should be the same in the mass spectra of the samples than in the mass spectra of the spiked samples with a tolerance of ±30% (see Figure S1 as an example).

Once the analytes were confirmed, they were then quantified in the samples. For this purpose, matrix-matched calibration curves for each honey sample were constructed in the working range, from 1 to 500 μg/kg (8 point calibration curve), as previously explained for validation in Section 2.5. The integration of the peak area signal was performed on the extracted ion chromatograms (EICs) of the product ion selected for quantification for each analyte (Table 1) using QuantAnalysis 2.2 software (Bruker Daltonics, Billerica, MA, USA). The content of the target analytes in the honey samples was calculated by using the average value obtained from the analysis of 3 replicates per sample.

### 2.7 Assessment of the Greenness of the Proposed Method

The eco-friendly properties of the microextraction procedure proposed using the μ-SPEed technique for the determination of PAs and TAs in honey samples were evaluated in terms of greenness using the Analytical Greenness Metric for Sample Preparation (AGREEprep),^39^ which is based on 10 consecutive steps of assessment that correspond to the 10 principles of green sample preparation. Moreover, it provides information about the strengths and weaknesses of the procedure.

### 2.8 Statistical Analysis

The statistical analysis of the optimization process was performed with SPSS 28.0.1.0 software, using student *t*-test for comparison of two means or one-way analysis of variance (ANOVA) and the Duncan posthoc multiple range test (significant differences at *p* ≤ 0.05) for the comparison of more than two means. On the other hand, the statistical analysis of honey samples was carried out with the MetaboAnalyst 5.0 web-based tool. The data were normalized (data cube root transformation and data autoscaling) and subjected to ANOVA using Fisher’s posthoc test. Differences were considered significant at *p* ≤ 0.05. Principal component analysis (PCA) and partial least-squares-discriminant analysis (PLS-DA) were used for multivariate statistical analysis to visualize differences or similarities among the sample profiles and identify the alkaloids that may indicate differences among the different honey samples analyzed. These analyses were performed by considering each sample as a different group. Hierarchical cluster analysis (HCA) was also performed using the PAs and TAs quantified in the honey samples by ANOVA and was constructed by Ward’s algorithm and Euclidean distance analysis to characterize the honey samples analyzed.

## 3 Results and Discussion

### 3.1 Development and Optimization of the Microextraction Procedure

Before microextraction, the honey samples were dissolved in 0.05 M H_2_SO_4_, which, according to the literature, has been frequently used as a solvent for this type of food sample (Table S2). According to the literature, the use of this acid helps to disintegrate and release PAs from the matrix, as well as decreasing the viscosity of the honey solution and avoiding clogging problems in the extraction cartridges.^9,20^ Thus, it was used to avoid overpressure problems during the aspiration-dispensing process in the μ-SPEed that may affect the analytical performance of the method regarding reproducibility as well as affecting the shelf life of the cartridges.

For the development of the microextraction procedure by μ-SPEed, some parameters (type of sorbent, washing step, number of extraction cycles, and elution volume) were evaluated to establish the optimal conditions for the method’s performance. These parameters and conditions were selected based on the extraction efficiency achieved in terms of recovery assays. For this purpose, the honey samples were spiked with a standard solution at a known concentration of the target analytes (50 μg/kg) and then subjected to the sample preparation procedure. The recovery was calculated by comparing the results obtained with the ones achieved using a simulated sample, as previously explained in Section 2.5. All recovery trials were performed in triplicate for each condition tested.

The type of sorbent was the first parameter to be evaluated. According to previous works, C18 and PS/DVB sorbents have proved to be effective for the extraction of PAs and TAs.^46,47^ Hence, these two types of microcartridges were evaluated, and based on these works, the following preliminary μ-SPEed conditions were set: 2 × 100 μL aspiration-dispense cycles of water and 2 × 100 μL aspiration-dispense cycles of H_2_SO_4_ 0.05 M for conditioning and equilibration of the sorbent, 5 × 100 μL aspiration-dispense cycles of sample, one aspiration-dispense cycle of 100 μL of water for the washing step, and 2 × 100 μL aspiration-dispense cycles of MeOH for elution (final elution volume = 200 μL). In μ-SPEed, two different operation modes are possible for sample loading: draw-eject mode (the sample volume aspirated is dispensed in the same vial containing the sample) or extract-discard mode (the sample volume aspirated is dispensed in a waste vial is different from the sample vial). In this work, the extract-discard operation mode was selected to achieve higher preconcentration of the analytes. Likewise, these conditions were also tested without carrying out a washing step before elution to determine if it affected the extraction efficiency of the analytes. The results revealed that the washing step has a big effect in some analytes when both types of cartridges were used, but especially in the case of the PS/DVB sorbent (Figure S2). In the C18 sorbent, the washing step mainly affected the extraction efficiency of the most polar compounds (intermedine, europine, and lycopsamine), whereas no significant differences were observed in the retention of the other analytes (Figure S2a). Conversely, in the PS/DVB sorbent, the washing step had a big effect on a higher number of analytes (intermedine, europine, lycopsamine, europine *N*-oxide, scopolamine, intermedine *N*-oxide, lycopsamine *N*-oxide, retrorsine, retrorsine *N*-oxide, and seneciphylline) (Figure S2b), which correspond to the first eluting compounds in the chromatographic analysis (Table 1). These results suggested that the washing step promoted the early elution of these compounds due to their polar characteristics and may indicate their weaker interaction with the PS-DVB as they are more easily eluted with a polar solvent. Therefore, the washing step should be omitted to avoid the loss of these analytes. Nonetheless, since the aim of the washing step is to eliminate matrix interferences, the signals achieved in the MS spectra with and without the washing step were compared for each analyte. No important differences were observed among the signals; therefore, it was decided to avoid this step. This could be due to the fact that very few mg of sorbent are used in μ-SPEed and a lot of pressure is generated during the dispense cycles, so the syringe is completely emptied, and no sample remains embedded in the sorbent. Consequently, very few matrix interferences are carried over during the elution step.

**Figure 1:**
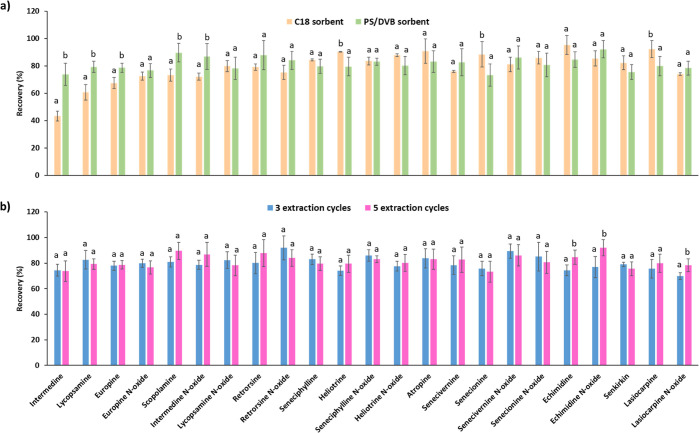
(a) Recovery values obtained with C18 and PS/DVB cartridges from the μ-SPEed analysis of a honey sample spiked with the analytes (50 μg/kg of each analyte) using the following extraction conditions: cartridge conditioning with 2 × 100 μL water and 2 × 100 μL 0.05 M sulfuric acid; 5 × 100 μL sample loading; and elution with 2 × 100 μL MeOH. (b) Recovery values obtained with PS/DVB cartridge from the μ-SPEed analysis of a honey sample spiked with the analytes (50 μg/kg of each analyte) using different extraction cycles for sample loading (3 and 5 extraction cycles) and the following extraction conditions: cartridge conditioning with 2 × 100 μL water and 2 × 100 μL 0.05 M sulfuric acid and elution with 2 × 100 μL MeOH. Same letters mean that there are no statistically significant differences (*p* > 0.05) and different letters mean that there are significant differences (*p* ≤ 0.05).

On the other hand, it was observed that under the conditions tested without a washing step, the PS/DVB sorbent generally provided better extraction efficiency than the C18 sorbent (Figure 1a). Good recovery values (>72%) were achieved with the C18 sorbent for all the analytes, except for the most polar ones (47% intermedine, 67% europine, and 63% lycopsamine). In contrast, with the PS-DVB sorbent, the recovery values of all the target analytes ranged from 73 to 92% (Figure 1a). Therefore, despite the fact that the interaction of the polar analytes with the PS/DVB may be weaker than with the C18 sorbent, as suggested from the results of the washing step, the affinity for the C18 sorbent is lower than for the PS-DVB sorbent in the case of the most polar analytes (intermedine, europine, and lycopsamine). This is reasonable because of the different types of interactions provided by the two sorbents. In the C18 sorbent, hydrophobic interactions mainly take place, so retention of very polar analytes may be less effective. In contrast, despite the hydrophobicity of the PS/DVB, which also has a nonpolar retention mechanism, this sorbent also presents aromatic groups in its polymeric structure that provide high selectivity for compounds with aromatic rings because of π–π interactions. For this reason, the PS/DVB sorbent may be more efficient in the retention of certain analytes compared to other hydrophobic sorbents, such as C18. Moreover, according to the manufacturer specifications (EPREP, Mulgrave, Victoria, Australia), the PS/DVB microcartridge presents a higher superficial area (300 Å) than the C18 sorbent (120 Å), so it may exhibit a higher loading capacity, enabling higher retention of the analytes. Therefore, according to the results obtained, the washing step was omitted, and the PS/DVB microcartridge was used in the following optimization trials.

After selecting the type of sorbent, different elution volumes (100, 200, and 300 μL) were evaluated using 5 sample extraction cycles without a washing step. It was observed that 100 μL of MeOH were not enough to completely elute all the analytes, since the recovery of 11 analytes out of 23 showed recovery values below 70% (ranging from 40 to 65%) (Figure S3). In contrast, no big differences were observed among 200 and 300 μL of MeOH, providing in both cases recovery values higher than 70% in all of the target analytes (Figure S3). Nonetheless, although good recoveries were obtained under these conditions, with the aim to minimize the use of organic solvents as well as the amount of sample and time required, the number of extraction cycles was reduced. Accordingly, the same procedure was carried out, but performing 3 extraction cycles instead of 5 and using 100 and 200 μL as elution volume (because as previously confirmed, increasing the volume to 300 μL did not show big differences compared to 200 μL). No significant differences were observed between 3 and 5 extraction cycles using 200 μL as the elution volume, achieving good recovery values for all the target analytes (Figure 1b). Likewise, it was observed that in the case of 3 extraction cycles, 100 μL of MeOH was enough to elute all the analytes and achieve suitable recovery values for method performance (recovery values ranging from 72 to 97%) (Figure S4). This is reasonable because with 3 extraction cycles, the analyte loading in the sorbent is lower than with 5 cycles, so a lower volume of solvent can be enough to elute all the analytes retained. Moreover, no significant differences were observed among 100 and 200 μL of MeOH when using 3 extraction cycles (Figure S4). Therefore, 3 extraction cycles and an elution volume of 100 μL of MeOH were selected for the final conditions. Thus, the overall microextraction procedure by μ-SPEed was as follows: PS-DVB sorbent, 3 extract-discard extraction cycles (3 × 100 μL of honey sample extract), and elution with 100 μL of MeOH without prior washing step. Under the final conditions selected, the overall μ-SPEed procedure took approximately 3 min per sample, using only 100 μL of organic solvent MeOH and 300 μL of diluted sample, which can be considered an ecofriendly and quick extraction method of PAs and TAs.

After the μ-SPEed procedure, the analytes were directly eluted in a chromatographic vial, and the final volume was checked prior to their injection in the chromatographic system. As explained in Section 2.4, the elution gradient of the chromatographic separation started with a high aqueous content (95% of water). This can sometimes be a problem when the sample injected in the system contains 100% MeOH because it can lead to peak broadening, especially in the case of the more polar analytes. However, this issue was investigated, and it was observed that with the chromatographic method employed, no problems were detected when injecting the sample extract in 100% MeOH (Figure S5). In addition, different injection conditions were tested, including a “sandwich injection” in which the analytes were injected between two volumes of the initial mobile phase, but the results obtained were the same than directly injecting the sample in 100% MeOH. One reason could be that the mobile phase gradient does not start with a 100% of the aqueous phase. Moreover, the first analyte was eluted at 6.3 min, so it is not at the beginning of the initial conditions but when the percentage of organic solvent is higher (approximately 25%) in the mobile phase. Therefore, since no chromatographic incompatibilities were detected, the sample extracts obtained from the μ-SPEed procedure were directly injected in the UHPLC–IT-MS/MS system.

### 3.2 Method Validation

The MS^2^ spectra and EICs of product ions obtained from spiked honey samples and standard solutions at the same concentration were compared to determine if other compounds in the sample matrix interfered in the detection of the target analytes (see Figures S1, S6, and S7). As it showed, no interfering peaks were observed at the retention time of the analytes, which was in all cases within the interval ±2.5%. In the case of those compounds that were isomers, two peaks were observed in their EIC at different times but with the same MS^2^ spectrum, such as the case of intermedine and lycopsamine, senecivernine, and senecionine, and their *N*-oxides (Figures S5–S7). The elution order of these isomers was determined by injecting them separately using individual standard solutions of these compounds when developing the chromatographic method. Regarding the MS^2^ spectra, the variations observed do not exceed 30%, and the fragment ratio was maintained both in the spectra of the spiked samples and in those of the standard solutions. Moreover, it was confirmed that the fragments obtained as product ions matched those obtained by direct infusion of the individual standard solutions (Table 1). Therefore, the selectivity of the method was confirmed.

Good lineal regression for all the analytes was achieved within the linear range evaluated based on the excellent correlation coefficients (*R*^2^) obtained, which were ≥0.999 in all cases, except for lasiocarpine (*R*^2^ = 0.998) (Table 2). The variation in the slope of the different matrix-matched calibration curves constructed in 3 different days was less than 30%, demonstrating good consistency. On the other hand, the results obtained from the slopes of both matrix-matched and solvent-based calibration curves revealed ME for some analytes (Table 2). Seventeen analytes out of 23 did not show ME, as their ME values were within the range of ±20%, so they could be quantified with solvent-based calibration curves instead of matrix-matched calibration curves. In contrast, 6 analytes showed ME values out of this range, so they must be quantified with matrix-matched calibration curves because they are influenced by the matrix. Only europine *N*-oxide was strongly affected by the matrix in a positive way (ME value > 50%), whereas the other 5 analytes only presented soft ME (values ranging between −50% < MEs < −20% and 50% > MEs > 20%). Accordingly, echimidine and senkirkine showed soft signal suppression, while lycopsamine, lycopsamine *N*-oxide, and heliotrine *N*-oxide showed soft signal increases (Table 2). Therefore, the sample preparation procedure proposed in this work provides satisfactory cleanup of matrix interference that helps to analyze and determine the target analytes in the honey samples.

**Table 2:** Analytical Parameters of the μ-SPEed Procedure Proposed and Coupled to UHPLC–IT-MS/MS for the Determination of Pyrrolizidine and Tropane Alkaloids in Honey Samples^a^

analyte	validation level (μg/kg)	recovery (% ± sd)	intra-day precision (RSD %)	inter-day precision (RSD %)	MDL (μg/kg)	MQL (μg/kg)	ME (%)	matrix-matched calibration curves (linearity, *R*^2^)
intermedine	5.00	75 ± 6	7	8	0.30	1.00	–0.1	*y* = 64,668*x*–307,385
	50.00	73 ± 7	6	10				0.999
	500.00	72 ± 6	8	8				
lycopsamine	5.00	94 ± 8	7	9	0.30	1.00	34	*y* = 26,855*x*–93,115
	50.00	83 ± 2	2	9				0.999
	500.00	97 ± 7	7	8				
europine	5.00	76 ± 7	8	9	0.30	1.00	19	*y* = 126,651*x*–885,656
	50.00	87 ± 7	5	8				0.999
	500.00	89 ± 9	8	10				
europine *N*-oxide	5.00	82 ± 6	7	7	0.30	1.00	67	*y* = 159,107*x*–1,095,242
	50.00	86 ± 2	2	4				0.999
	500.00	91 ± 7	10	8				
scopolamine	5.00	81 ± 6	8	15	0.30	1.00	–2	*y* = 106,454*x*–725,986
	50.00	86 ± 4	5	5				0.999
	500.00	89 ± 11	7	12				
intermedine *N*-oxide	5.00	76 ± 10	9	13	0.20	0.50	–11	*y* = 49,188*x*–222,854
	50.00	85 ± 8	5	10				0.999
	500.00	92 ± 9	8	10				
lycopsamine *N*-oxide	5.00	77 ± 6	8	12	0.20	0.50	43	*y* = 47,255*x*–172,570
	50.00	91 ± 8	8	9				0.999
	500.00	88 ± 9	9	10				
retrorsine	5.00	75 ± 8	9	10	0.30	1.00	–8	*y* = 38,948*x*–129,069
	50.00	88 ± 1	2	10				0.999
	500.00	74 ± 7	10	11				
retrorsine *N*-oxide	5.00	99 ± 8	3	8	0.30	1.00	–16	*y* = 3394*x* + 162,044
	50.00	93 ± 4	5	10				0.999
	500.00	82 ± 12	13	14				
seneciphylline	5.00	78 ± 9	10	11	0.30	1.00	1	*y* = 30,949*x* + 32,870
	50.00	87 ± 2	2	5				0.999
	500.00	88 ± 6	7	9				
heliotrine	5.00	90 ± 10	4	12	0.20	0.50	–9	*y* = 85,021*x*–584,830
	50.00	80 ± 2	3	5				0.999
	500.00	100 ± 6	6	6				
seneciphylline *N*-oxide	5.00	81 ± 10	9	12	0.23	0.75	12	*y* = 67,640*x*–312,550
	50.00	88 ± 4	5	5				0.999
	500.00	84 ± 9	11	12				
heliotrine *N*-oxide	5.00	86 ± 10	11	12	0.30	1.00	24	*y* = 52,576*x*–236,661
	50.00	81 ± 10	5	12				0.999
	500.00	92 ± 9	10	12				
atropine	5.00	89 ± 8	8	15	0.30	1.00	–3	*y* = 102,383*x*–451,267
	50.00	97 ± 10	9	10				0.999
	500.00	89 ± 9	10	12				
senecivernine	5.00	80 ± 12	10	15	0.30	1.00	12	*y* = 116,112*x*–580,380
	50.00	81 ± 10	9	12				0.999
	500.00	78 ± 10	12	12				
senecionine	5.00	85 ± 8	8	9	0.30	1.00	–12	*y* = 30,098*x*–125,140
	50.00	84 ± 5	5	8				0.999
	500.00	91 ± 7	6	8				
senecivernine *N*-oxide	5.00	87 ± 6	8	15	0.30	1.00	4	*y* = 8233*x* + 22,132
	50.00	89 ± 7	6	8				0.999
	500.00	87 ± 7	9	10				
senecionine *N*-oxide	5.00	83 ± 3	5	15	0.30	1.00	0.6	*y* = 24,566*x* + 2014
	50.00	97 ± 5	5	13				0.999
	500.00	87 ± 11	10	13				
echimidine	5.00	85 ± 11	4	13	0.12	0.40	–23	*y* = 135,889*x*–535,274
	50.00	83 ± 3	3	6				0.999
	500.00	75 ± 9	4	11				
echimidine *N*-oxide	5.00	86 ± 11	7	11	0.30	1.00	18	*y* = 18,090*x* + 56,670
	50.00	79 ± 4	5	11				0.999
	500.00	93 ± 8	7	9				
senkirkine	5.00	82 ± 6	5	7	0.24	0.80	–36	*y* = 36,795*x*–163,195
	50.00	85 ± 5	2	6				0.999
	500.00	89 ± 8	9	9				
lasiocarpine	5.00	82 ± 8	6	10	0.13	0.42	–17	*y* = 13,435*x* + 7307
	50.00	81 ± 10	10	13				0.998
	500.00	78 ± 9	12	15				
lasiocarpine *N*-oxide	5.00	93 ± 10	10	11	0.20	0.50	–18	*y* = 411,122*x* + 1,088,550
	50.00	72 ± 6	4	8				0.999
	500.00	88 ± 8	8	9				

a Recovery: mean recovery obtained from nine samples (*n* = 9) spiked with the analytes at the different validation levels and subjected to the proposed extraction procedure; intra-day precision: six replicate extracts (*n* = 6) from a honey sample spiked with the analytes at the different validation levels and analyzed on the same day; inter-day precision: three replicate extracts from a honey sample spiked with the analytes at the different validation levels and analyzed throughout three different days (*n* = 9); MDL: method detection limit; MQL: method quantification limit; ME: matrix effect.

The method also provided good sensitivity, enabling detection of the analytes even at lower concentrations than the ones set in the legislation for these compounds in other foodstuffs. The MDLs and MQLs for the 23 alkaloids ranged from 0.12 to 0.30 μg/kg and 0.40 to 1.00 μg/kg, respectively (Table 2). The concentrations estimated for the MDLs were confirmed by spiking blank honey samples at these concentrations and verifying that the peaks of the analytes are still clearly visible in the EICs.

The accuracy was also successfully evaluated at the three concentration levels proposed, as the recovery values obtained were within the range set in the validation guidelines (70–120%). As can be observed, the overall average recovery values ranged from 72 to 100% in three validation levels (Table 2), confirming enough reliability of the method. Likewise, Table 2 also shows the method’s precision in terms of intra-day repeatability and inter-day reproducibility at the three validation levels. As can be observed, RSD values were ≤13 and ≤15% for intra-day and inter-day precision, fulfilling the criteria set in the validation guidelines (RSD values should be ≤20%). Therefore, the method developed and proposed in this work shows good analytical performance as the analytical parameters fully accomplished the validation guidelines,^49−51^ so it can be reliably applied to the microextraction and analysis of PAs and TAs in honey samples.

### 3.3 Greenness Evaluation of the Method

**Figure 2:**
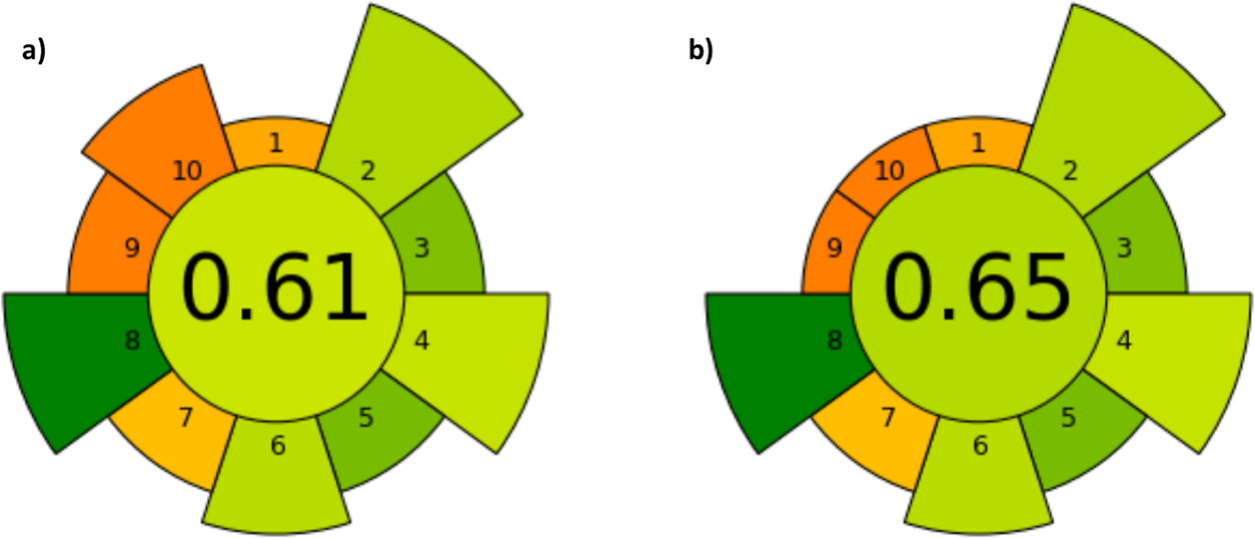
Results of the AGREEprep assessment of the μ-SPEed procedure for the determination of pyrrolizidine and TAs in honey samples after applying (a) the default weights and (b) modifying the weight of criteria nos. 9 and 10 in the tool to the lowest value (weight value = 1).

The eco-friendly properties of the analytical methodology proposed are shown in Figure 2. As previously indicated in Section 2.7, the greenness of the method was assessed with the AGREEprep tool using the default weights established. The overall sample preparation greenness performance is indicated by the inner circle color (based on traffic light colors) and the assigned overall score. Overall values can range from 0 to 1, with score 1 being the greenness performance. As it can be observed, the method proposed for the analysis of PAs and TAs in honey samples is scored with a value of 0.61 and the inner circle color is light green (Figure 2a), which indicates that the method can be considered a green analytical procedure. The justification for each input used to assign the AGREEprep score is included in Table S3 and Figures S8 and S9 showing the reports obtained. Moreover, around the circle, there are 10 numbers that correspond to each performance criteria. The length of each number indicates the weight assigned to each criterion and the color visualizes the criterion performance. Thus, it is a quick and visual way to identify the strong and weak points of the procedure, as well as the aspects to be improved. Accordingly, for the method proposed, the weak points are the criteria: 1 (favor in situ sample preparation), 7 (integrate steps and promote automation), 9 (choose the greenest possible postsample preparation configuration for analysis), and 10 (ensure safe procedures for the operator) (Figure 2a). One way to improve it would be to increase the automation of the systems and promote online procedures. In fact, the method here proposed with μ-SPEed can be scaled to an automatic system by using the ePrep Sample Preparation Workstation (EPREP, Australia). However, this would lead to higher energy consumption and require an important financial investment. Another important aspect to be improved could be replacing MeOH for a green solvent, but the elution efficiency should be confirmed. Nonetheless, the amount of MeOH used per sample is minimal (100 μL), but this solvent has 3 hazard pictograms that strongly penalize in the criterion no. 10 of the AGREEprep tool. Another criterion that hardly penalizes is no. 9, since the tool promotes direct MS analysis without performing chromatographic separation due to the mobile phase consumption and its composition in organic solvents. However, to perform a multicomponent analysis, the chromatographic separation is needed, especially when there are isomers in the same samples, as is the case. Based on this justification, if these criteria (nos. 9 and 10) are modified to a weight lower in the tool, the method would lead to an overall score of 0.65 (Figure 2b). Nonetheless, according to the other criteria, the method fulfills the principles of miniaturized sample preparation, as it is quick, uses low amounts of samples and reagents, minimizes the production of waste, and has very low energy consumption.

On the other hand, comparing the proposed method with other works published in the literature that have determined PAs and/or TAs in honey samples, it can be observed that almost all of them have used conventional sample preparation techniques such as SPE, QuEChERS, or even salting-out assisted LLE (SALLE) (Table S2). As is evidenced, these procedures usually purify high volumes of sample extracts, require sorbent materials with an average amount range of 50–500 mg, and use important volumes of organic solvents (of the order of mL), which in most of the cases are then evaporated to dryness, involving significant energy consumption. In addition, in SPE, a vacuum pump is needed, while μ-SPEed does not require one and the digital syringe can be taken to the sampling place, so it allows much easier sample preparation anywhere. Moreover, in the QuEChERS and SALLE procedures, multiple steps are needed, including centrifugation processes and the addition of multiple reagents (salts and/or cleanup sorbents), leading to time-consuming protocols and to a high generation of waste. In consequence, many of these strategies do not meet the criteria established by the Green Analytical Chemistry (GAC) to develop green and sustainable analytical methods. Accordingly, the miniaturized μ-SPEed techniques provide multiple advantages over these conventional sample preparation procedures, highlighting their ability to develop quick extractions using very few μL of sample and solvents and low sorbent amounts, providing high extraction efficiency and the possibility to achieve a high concentration factor in the same procedure without needing a subsequent evaporation step. Regarding miniaturized procedures, to the best of our knowledge, there is only one work in the literature that has applied a microextraction strategy for the determination of PAs in honey samples.^22^ In this work, the DLLME technique is used for the extraction of 9 PAs from honey samples. Nonetheless, as it can be observed, although it is considered a green strategy because low amounts of organic solvents are required, several steps need to be performed (e.g., centrifugation and evaporation). Moreover, higher sample volumes, organic solvent volumes, and reagent amounts are used compared to the μ-SPEed procedure proposed in this work (Table S2). On the other hand, some authors have omitted the sample preparation step.^29,30^ In these works, the honey sample is directly dissolved in water or an aqueous ammonium hydroxide solution and injected into the chromatographic system after filtration and/or centrifugation. However, due to the complexity of food samples, it is convenient to perform a sample preparation procedure to purify the extracts before the analysis and avoid injecting them directly into the system because matrix interferences can foul the ionization source of the mass spectrometer detector and decrease sensitivity. In addition, this also helps to extend the life of the chromatographic column, contributing also to reducing the cost and consumption of solvents for its washing. Moreover, sample preparation is interesting in the analysis of contaminants because it allows preconcentration of the analytes and achieves the sensitivity set by legislation.

On the other hand, some previous works have already developed methodologies for the simultaneous analysis of PAs and TAs in honey samples.^19,31,32^ However, the number of total analytes analyzed in these articles is lower than the number of alkaloids proposed by our group (Table S2). Moreover, in these works, higher amounts of sorbents and solvents are used, and the procedures require more time and steps (Table S2). Thus, regarding the literature, the method proposed by μ-SPEed shows important improvements, especially in terms of greenness, highlighting its ability to develop easier and quicker extractions using very few μL of sample and solvents, providing high extraction efficiency and the possibility to achieve a high concentration factor in the same procedure without needing a subsequent evaporation step. Therefore, it can be concluded that μ-SPEed can be used as an alternative and potential microextraction technique to develop quick, green, sensitive, selective, and cost-effective analytical procedures within the GAC principles and under the commitment of the SDGs.

### 3.4 Analysis of Honey Samples

**Figure 3:**
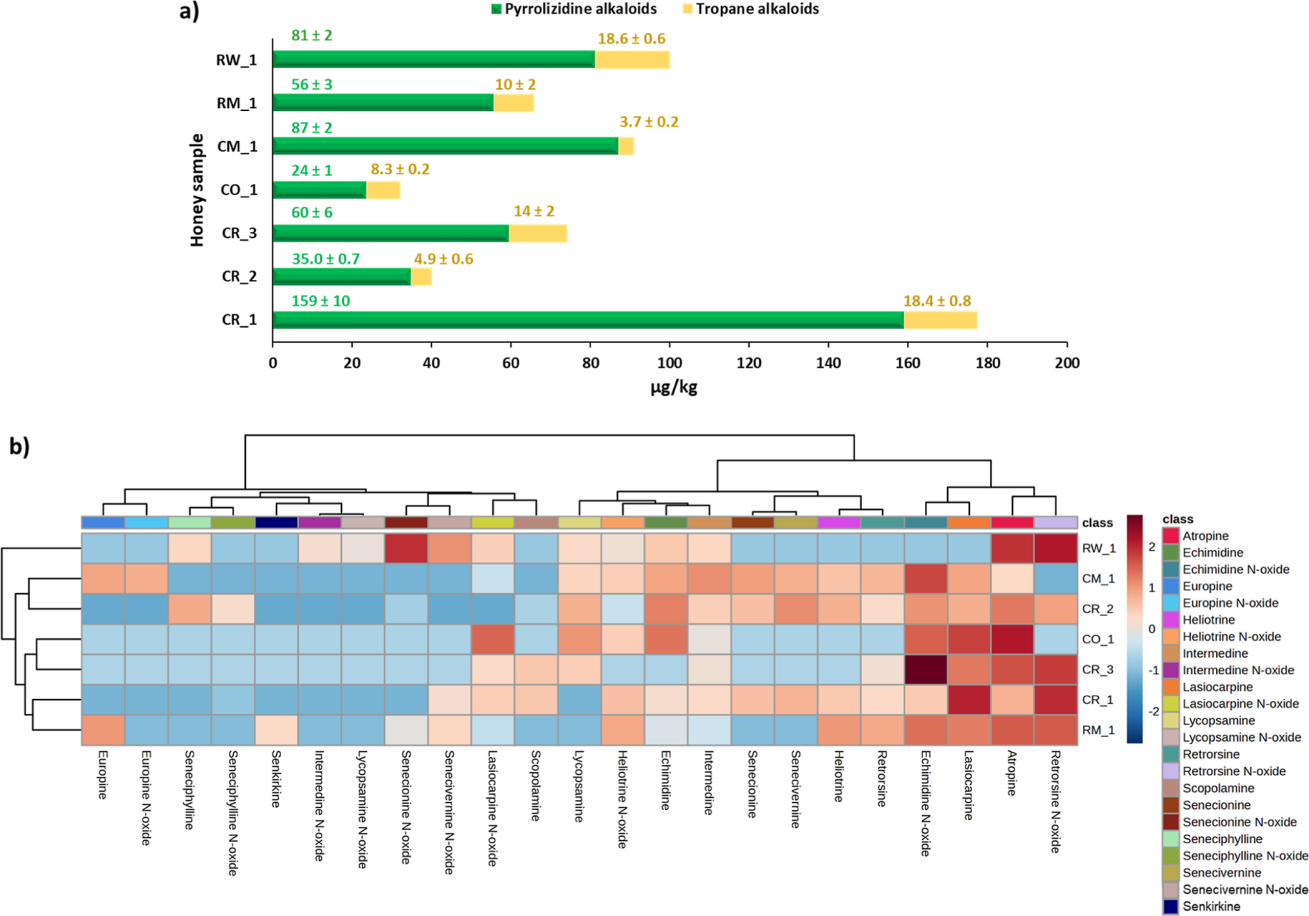
(a) Total content (μg/kg) of pyrrolizidine and TAs in honey samples and (b) HCA and heat map from the dataset of the individual target alkaloids found in the different honey samples analyzed. The columns represent the analytes and the rows the samples analyzed. The color gradient ranging from dark blue through white to dark red indicated the relationship among the analytes in each sample, which represents low, middle, and high abundance of the analytes. The resulting dendrogram associated with the heat map was generated by Ward’s algorithm and Euclidean distance analysis. Sample information codes are provided in Table S1.

The μ-SPEed procedure developed was applied to the analysis of 7 honey samples (Table S1). The quantification of the PAs and TAs detected and confirmed on the samples was performed by constructing matrix-matched calibration curves for each sample within the linear range validated. Figure 3a shows the total content of PAs and TAs in the honey samples analyzed. As can be observed, 100% of the samples analyzed showed contamination with these alkaloids, although not all of the target analytes were always found in the samples. The orange blossom honey (CO_1) was the least contaminated, while one of the rosemary honeys (CR_1) presented the highest contamination value (Figure 3a). The level of TAs found in the honey samples ranged from 3.7 to 18.6 μg/kg (Figure 3a). These values agree with the concentration of TAs determined by other authors in honey samples (Table S2). RW_1 and CR_1 were the samples with the highest contamination of TAs, mainly atropine, while CM_1 and CR_2 were the samples least contaminated with these alkaloids (Figure 3). On the other hand, the contamination levels of PAs in the honey samples ranged from 24 to 159 μg/kg (Figure 3a). According to the literature, these concentration values are within the contamination range reported in previously published works (Table S2).

**Figure 4:**
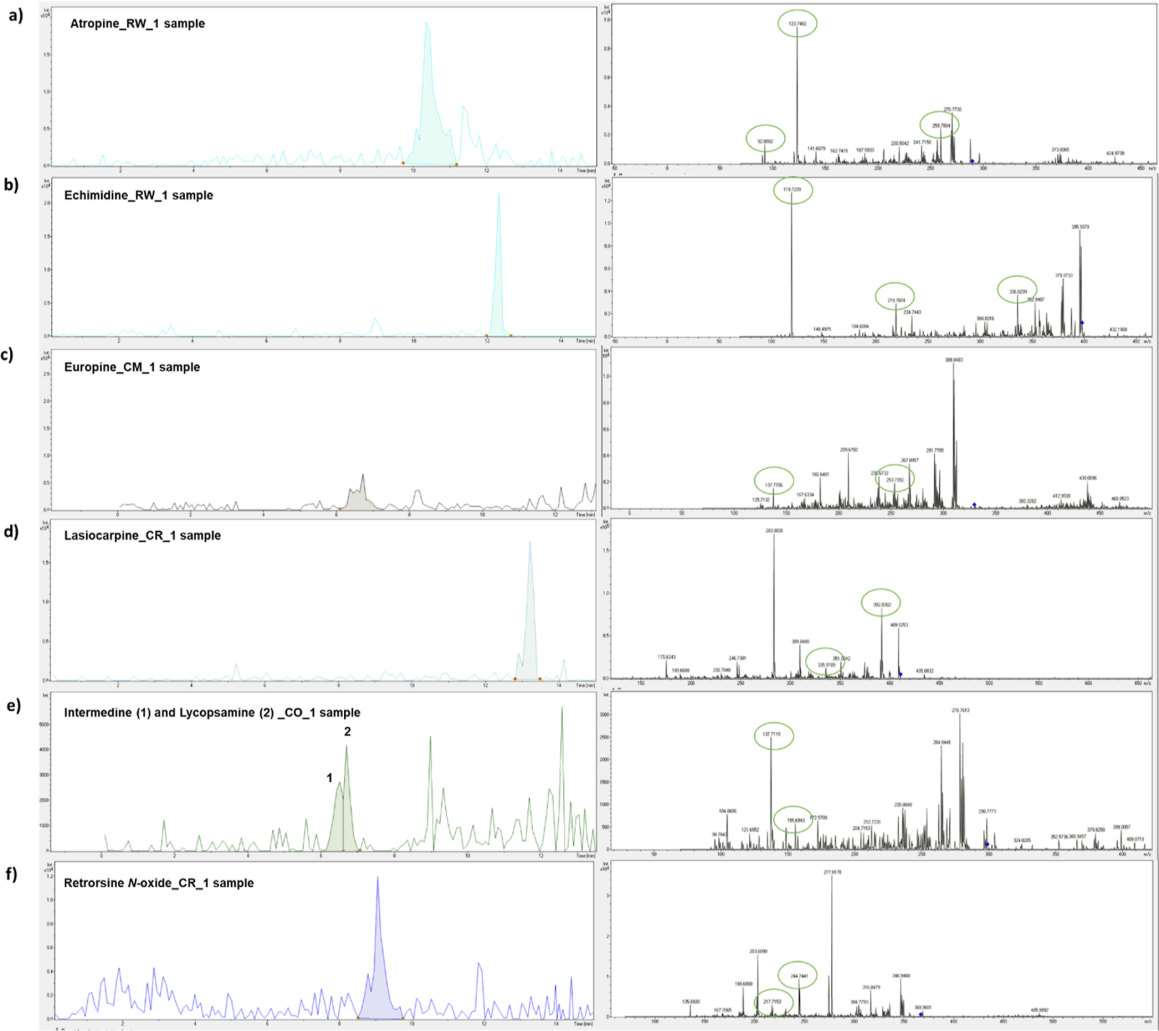
EICs of product ions used for quantification and MS^2^ of (a) atropine in sample RW_1, (b) echimidine in sample RW_1, (c) europine in sample CM_1, (d) lasiocarpine in sample CR_1, (e) intermedine and lycopsamine in sample CO_1, and (f) retrorsine *N*-oxide in sample CR_1 after μ-SPEed extraction and UHPLC–IT-MS/MS analysis. Green circles indicated the product ion set for quantification and confirmation of the target analytes. Sample information codes are provided in Table S1.

The contamination profile of the different honey samples based on the individual analysis of PAs and TAs quantified in them is shown in Figure 3b by means of a HCA. Regarding TAs, atropine was found in all the honey samples analyzed, highlighting its concentration in sample RW_1 (Figure 4). In contrast, scopolamine was only identified in the rosemary honey samples. Regarding PAs, very different contamination patterns were observed among the samples analyzed. In general, in all the honey samples, PAs belonging to the three main families (heliotrine-type, lycopsamine-type, and senecionine-type) were found (Figure 4), except in the orange blossom honey sample (CO_1), which did not show senecionine-type PAs. According to the Mediterranea flora, the source of heliotrine-type PAs (mainly lasiocarpine and heliotrine) could be *Heliotropium* sp., lycopsamine-type PAs (mainly echimidine, lycopsamine, and intermedine) could be associated with *Echium* spp., whereas senecionine-type PAs (mainly retrorsine, senecionine, senecivernine, and seneciphylline) will be linked to the predominance of Senecio species.^9,52^ Overall, retrorsine *N*-oxide, lasiocarpine, echimidine, and echimidine *N*-oxide were the PAs that had the greatest weight in the contamination of the samples (Figure 3b). Conversely, it was observed that europine was only found in the multifloral honey samples, especially in the case of the honey sample from Israel (CM_1) (Figures 3b and 4). This result agrees with previous data reported for honey from Greece, in which a significant presence of europine was detected in retail honey samples collected in this area,^52^ which is geographically close to Israel. Likewise, senkirkine was detected only in sample RM_1. Contamination with senecionine-type PAs clearly predominated in sample RW_1, highlighting the contribution of retrorsine *N*-oxide and senecionine *N*-oxide. On the other hand, very different PA patterns were observed among the rosemary honey samples. For instance, in sample CR_1 heliotrine- and senecionine-type PAs predominated, highlighting the occurrence of lasiocarpine and retrorsine *N*-oxide, respectively, while lycopsamine-type PAs (mainly echimidine and echimidine *N*-oxide) were found to a lesser extent in this sample. In contrast, lycopsamine-type and senecionine-type PAs predominated in sample CR_2, while sample CR_3 mainly showed lycopsamine- and heliotrine-type PAs, and only retrorsine and retrorsine *N*-oxide as senecionine-type PAs. In order to evaluate the differences and similarities among the contamination profiles of the samples, a PCA and a PLS-DA were performed as a multivariate analysis (Figure S10). The PCA is an unsupervised method that allows to visualize differences and similarities among samples and identify the significant variables that contribute to these discrepancies. Figure S10a shows the PCA score plot from the honey samples analyzed. The PC1 and PC2 variances were 33.2 and 20.9%, respectively, representing 54.1% of the total PAs and TAs variability of the data, which provides good differentiation of the honey samples. As it can be observed, CR_2, CR_3, RM_1, and CM_1 were projected in PC1 and PC2 negative quadrants, indicating similarities among these samples. In contrast, CR_1 was the only sample projected in PC1 negative and PC2 positive quadrants, while RW_1 was projected in PC1 and PC2 positive quadrants and CO_1 in PC1 positive and PC2 negative quadrants, suggesting significant differences among these samples. The PLS-DA was carried out as a supervised clustering method, and according to the previous PCA analysis, the results obtained revealed good discrimination among the samples (Figure S10b). A total variance of 46.8% was obtained by the first two principal components from the PLS-DA. Moreover, the statistical contribution of each analyte in the projection used in the PLS was evaluated through a variable importance in projection (VIP) score (Figure S10c). A variable with a VIP score of ≥1 can be considered important in a given model. Accordingly, 11 target analytes (10 PAs and 1 TAs) presented a VIP score ≥1 (Figure S10c). Thus, senecionine *N*-oxide, atropine, senecionine, senecivernine, europine *N*-oxide, intermedine *N*-oxide, lycopsamine *N*-oxide, senecivernine, retrorsine *N*-oxide, seneciphylline, and echimidine were the most relevant compounds and the ones with the greatest discriminatory power to characterize the contamination of the honey samples analyzed.

## Supporting Information Available

Information of the honey samples analyzed; comparison of the proposed μ-SPEed method with sample preparation methods already published for the determination of PAs and/or TAs in honey samples within the last 5 years (2018–2023); EICs and mass spectra of heliotrine in honey sample spiked with a standard solution at a concentration of 25 μg/kg and in a honey sample nonspiked and naturally contaminated with heliotrine; recovery values obtained using C18 and PS/DVB μ-SPEed cartridges from the analysis of honey samples spiked with the target analytes (50 μg/kg of each analyte) performing and not performing a washing step during the μ-SPEed procedure before elution; recovery values obtained with PS/DVB μ-SPEed cartridges from the analysis of honey samples spiked with the target analytes (50 μg/kg of each analyte) performing 5 and 3 extraction cycles and using different elution volumes of MeOH; EICs and mass spectra of intermedine, lycopsamine, and europine in a standard solution of 500 μg/L using the chromatographic method; input used to assign AGREEprep scores for μ-SPEed-UHPLC–IT/MS/MS method; EICs of product ions used for quantification and mass spectra of tropane, pyrrolizidine, and pyrrolizidine *N*-oxides alkaloids in spiked honey samples and standard solutions at the same concentration level (60 μg/L); evaluation report of the AGREEprep assessment of the method proposed using μ-SPEed for the determination of pyrrolizidine and TAs in honey samples after applying the default weights of the tool and modifying the weight of criteria no. 9 and 10 in the tool to the lower value; and multivariate statistical analysis using PCA and partial least-squares-discrimination analysis of the pyrrolizidine and TAs determined in the honey samples analyzed (PDF)
