# Supplementary material for: Miniaturized Analytical Strategy Based on μ-SPEed for Monitoring the Occurrence of Pyrrolizidine and Tropane Alkaloids in Honey
Source: J Agric Food Chem. 2023 Dec 18;72(1):819–32. doi: 10.1021/acs.jafc.3c04805 (PMC10786043; doi:10.1021/acs.jafc.3c04805)
Supplement: Supplementary file 1 — jf3c04805_si_001.pdf [file jf3c04805_si_001.pdf]

Supplementary material

A miniaturized analytical strategy based on µSPEed® for monitoring the occurrence of pyrrolizidine and tropane alkaloids in honey

Natalia Casado†, Sonia Morante-Zarcero†, Isabel Sierra† *

†Departamento de Tecnología Química y Ambiental, E.S.C.E.T, Universidad Rey Juan Carlos, C/ Tulipán s/n, 28933 Móstoles, Madrid, Spain

* Corresponding author: Tel.: (+34) 914887018; fax: (+34) 914888143.

E-mail addresses: natalia.casado@urjc.es; sonia.morante@urjces; isabel.sierra@urjc.es

Table S1. Information of the honey samples analyzed.

Sample code	Type of honey (Botanical origin)	Geographical origin
CR_1	Commercial rosemary monofloral honey	Spain
CR_2	Commercial rosemary monofloral honey	Spain
CR_3	Commercial rosemary monofloral honey	Spain
CO_1	Commercial orange blossom monofloral honey	Spain
CM_1	Commercial multifloral honey	Israel
RM_1	Retail multifloral honey	Spain
RW_1	Retail woodland honey	Spain

Table S2. Comparison of the proposed µSPEed® method with sample preparation methods already published for the determination of pyrrolizidine alkaloids and/or tropane alkaloids in honey samples within the last 5 years (2018-2023).

Number of target alkaloids[a]	Sample preparation technique	Sample amount and treatment before extraction	Amount of solvents and reagents used in sample preparation	Time estimation per sample preparation[b]	Range of PAs and TAs found in honey samples	Ref
9 PAs and 2 TAs	QuEChERS	1.5 g dissolved in 10 mL 0.1 M H_2SO_4 + 0.5 g Zinc dust	QuEChERS salts: 4.9 g $MgSO_4$, 1 g trisodium citrate dehydrate, 0.5 g disodium hydrogen citrate sesquihydrate and 1 g NaCl)<br>QuEChERS sorbents: 150 mg PSA<br>10 mL 0.1 M H_2SO_4<br>0.5 g of zinc dust<br>10 mL ACN<br>1 mL ACN/0.1% formic acid in water (13:87 v/v).	1 h 40 min pre-QuEChERS<br>25 min QuEChERS + evaporation to dryness of 6 mL	Total contents not reported.<br>Atropine: 1.4 to 3.8 µg/kg<br>Echimidine: 0.4 to 3.3 µg/kg<br>Lycopsamine: 0.2 to 74.7 µg/kg	[19]
Total PAs expressed as Retronecine and Heliotrine derivattives	SPE	10 g dissolved in 20 mL 0.15 M HCl + 2 g Zinc dust	SPE cartridges: 500 mg MCX sorbent<br>9 mL MeOH<br>29 mL 0.15 M HCl<br>12 mL water<br>12.1 mL MeOH<br>9 mL ethyl acetate<br>12 mL ethyl acetate/MeOH/ammonia solution/triethylamine (8/2/0.1/0.1, v/v)	1 h 30 min pre-SPE<br>82 min SPE + 2 evaporation to dryness steps + reduction of alkaloids	1.0 - 64.1 µg/kg	[20]
12 PAs	SPE	1 g dissolved in 3 mL 0.05 M H_2SO_4	SPE cartridges: 150 mg SCX sorbent<br>7 mL 0.05 M H_2SO_4<br>4 mL MeOH<br>5 mL 2.5% ammonium hydroxide in MeOH (v/v)	40 min pre-SPE 46 min SPE<br>10 min post-SPE	1.4 – 14.2 µg/kg	[21]

			0.5 mL ACN/water (5/95, v/v)			
9 PAs	DLLME	10 g dissolved in 100 mL acidified water (0.25<br>M acetic acid)	100 mL acidified water (0.25 M acetic acid)<br>1.25 g of NaCl<br>500 µL of $CHCl_3$<br>500 µL of iPrOH<br>0.125 mL of MeOH/water 2/8 v/v	3 min pre-DLLME<br>5 min DLLME + evaporation to dryness of 1 mL	0.2 - 17.5 µg/kg	[22]
5 PAs	QuPPE	5 g dissolved in 10 mL water + 10 mL 1% formic acid in MeOH	10 mL water<br>10 mL 1% formic acid in MeOH	1 min pre- QuPPE<br>6 min QuPPE	43 -75 µg/kg	[23]
17 PAs	SPE	10 g dissolved in 30 mL 0.05 M H_2SO_4/MeOH (85/15, v/v)	SPE cartridges: MCX sorbent<br>30 mL 0.05 M H_2SO_4/MeOH (85/15, v/v)<br>6 mL MeOH<br>3 mL water<br>3 mL 0.05 M H_2SO_4<br>6 mL 15% ammoniated MeOH<br>0.1 mL water/ACN (85/15, v/v)	5 min pre-SPE 48 min SPE + evaporation to dryness of 6 mL	0.2 – 281.1 µg/kg	[24]
25 PAs	SPE	10 g dissolved in 30 mL 0.05 M H_2SO_4	SPE cartridges: 500 mg SCX sorbent<br>11 mL MeOH<br>35 mL 0.05 M H_2SO_4<br>6 mL water<br>10 mL 2.5% ammonia in MeOH<br>1 mL 10% MeOH	50 min pre-SPE 62 min SPE + evaporation to dryness of 10 mL	0.1 - 3313 µg/kg	[25]
2 PAs	SPE	20 g dissolved in 20 mL 0.05 M H_2SO_4	SPE cartridges: 200 mg SCX sorbent<br>20 mL 0.05 M H_2SO_4<br>4 mL MeOH<br>2 mL 0.1% formic acid<br>2 mL 0.05% formic acid<br>9 mL 0.1% ammoniated MeOH	10 min pre-SPE<br>37 min SPE + evaporation to dryness of 9 mL	-	[26]

9 TAs	QuEChERS	2.5 g dissolved in 10 mL of MeOH/water/ formic acid (75/25/0.4, v/v/v)	QuEChERS salts: 0.3 g $MgSO_4$,<br>10 mL of MeOH/water/ formic acid (75/25/0.4, v/v/v)<br>QuEChERS sorbents: 50 mg GBC	51 min	27 µg/kg	[27]
2 TAs	LLE	5 g dissolved in 10 mL concentrated sodium acetate solution + 10 mL ACN	10 mL concentrated sodium acetate solution<br>10 mL ACN	45 min	0.012 – 0.024 µg/kg	[28]
27 PAs	.	0.5 g dissolved in 10 mL of 6.5 mmol aqueous ammonium hydroxide solution	10 mL of 6.5 mmol aqueous ammonium hydroxide solution	10 min	Up to 141.8 µg/kg	[29]
8 PAs	-	2 g dissolved in 5 mL water	5 mL water<br>0.8 mL 0.1% formic acid/ACN containing 0.1% formic acid (98/2, v/v)	40 min	49.3 – 623 µg/kg	[30]
15 PAs and 2 TAs	SPE	10 g dissolved in 20 mL 0.05 M H_2SO_4 + 1 g Zinc dust	SPE cartridges: 500 mg MCX sorbent<br>32 mL 0.05 M H_2SO_4<br>1 g Zinc dust<br>32.2 mL MeOH<br>10.2 mL water<br>6 mL ethyl acetate<br>10 mL ethyl acetate/MeOH/ACN/ammonia solution/triethylamine (8/1/1/0.3/0.1, v/v)	24 h 40 min pre-SPE<br>82 min SPE + evaporation to dryness of 10 mL	2.2 – 147 µg/kg	[31]
4 PAs and 1 TAs	QuEChERS	1 g dissolved in 1 mL of water and 5 mL ACN	QuEChERS salts: 1 g NaCl<br>QuEChERS sorbents: 50 mg PSA<br>1 mL water	13 min + evaporation to dryness of 6 mL	-	[32]

30 PAs	QuEChERS	2 g dissolved in 20 mL 50% ACN containing 1% formic acid	5 mL ACN<br>0.5 mL ACN/water (5/95, v/v)<br>QuEChERS salts: 4 g $MgSO_4$, 1 g trisodium citrate dehydrate, 0.5 g disodium hydrogen citrate sesquihydrate and 1 g NaCl)<br>20 mL 50% ACN containing 1% formic acid<br>0.3 mL 1% formic acid	96 min + evaporation to dryness of 0.2 mL	Up to 74 µg/kg	[33]
9 PAs	SALLE	25 g dissolved in 100 mL water	100 mL water<br>$MgSO_4$ to achieve concentration 1 M<br>Na_2SO_4 to achieve concentration 1.5 M<br>1.5 mL ACN<br>0.5 mL water/MeOH (7/3, v/v)	3 min pre-SALLE<br>6 min SALLE + evaporation to dryness of 1.5 mL	Up to 37.3 µg/kg	[34]
28 PAs	SALLE	25 g dissolved in 100 mL water	100 mL water<br>$MgSO_4$ to achieve concentration 1 M<br>Na_2SO_4 to achieve concentration 1.5 M<br>2 mL ACN<br>0.125 mL water/MeOH (7/3, v/v)	18 min pre-SALLE<br>11 min SALLE + evaporation to dryness of 2 mL	-	[35]
4 PAs	SPE	10 g dissolved in 100 mL water	SPE cartridges: 30 mg sulfonated halloysite nanotubes<br>100 mL water<br>1 mL 0.03 mM formic acid<br>2 mL 0.1 M ammonium formate in MeOH<br>1 mL 100 µg L^{-1} caffeine solution in dimethylsulfoxide/MeOH (1/1, v/v)	5 min pre-SPE<br>8 min SPE + evaporation to dryness of 2 mL	-	[36]

4 PAs	SPE	4 g dissolved in 40 mL 0.05 M formic acid	SPE cartridges: 50 mg organosilyl-sulfonated halloysited<br>40 mL 0.05 M formic acid<br>1 mL 0.04 mM formic acid<br>2 mL MeOH/water (1:1, v/v)<br>2 mL 100 mM ammonium formate in MeOH<br>1 mL 100 µg L^{-1} caffeine solution in dimethylsulfoxide/MeOH (1/1, v/v)	10 min pre-SPE<br>45 min SPE + evaporation to dryness of 2 mL	-	[37]
35 PAs	QuEChERS	2.5 g dissolved in 15 mL 0.1 M H_2SO_4 + 15 mL ACN	QuEChERS salts: 4 g $MgSO_4$, 1 g trisodium citrate dehydrate, 0.5 g disodium hydrogen citrate sesquihydrate and 1 g NaCl<br>15 mL 0.1 M H_2SO_4<br>15 mL ACN<br>1 mL water/MeOH ()5/5, v/v)	45 min pre-QuEChERS<br>40 min QuEChERS + evaporation to dryness	0.9 – 33.1 µg/kg	[38]
21 PAs and 2 TAs	µSPEed®	0.5 g dissolved in 2.5 mL 0.05 M H_2SO_4	µSPEed®: 4 mg PS/DVB sorbent<br>0.2 mL water<br>2.7 mL 0.05 M H_2SO_4<br>0.1 mL MeOH	15 min pre-µSPEed®<br>3 min µSPEed®	32 to 177 µg/kg	This work

[a] Pyrrolizidine and/or tropane alkaloids.

[b] For time estimation, a flow rate of 1 mL min^{-1} was considered in SPE when this information was not provided by authors.

ACN: acetonitrile; DLLME: dispersive liquid-liquid microextraction; GCB: graphitized carbon black; iPrOH: isopropylalcohol; LLE: liquid-liquid extraction; MCX: mixed-mode sorbent with cation exchange-reversed phase interactions; MeOH: methanol; PAs: pyrrolizidine alkaloids; PS/DVB: porous crosslinked polystyrene divinyl benzene; PSA: primary secondary amine; QuEChERS: quick, easy, cheap, effective, rugged, and safe; QuPPE: simplified methanol extraction; SALLE: salting-out assisted liquid–liquid extraction; SCX: strong cation exchange sorbent; SPE: solid-phase extraction; TAs: tropane alkaloids

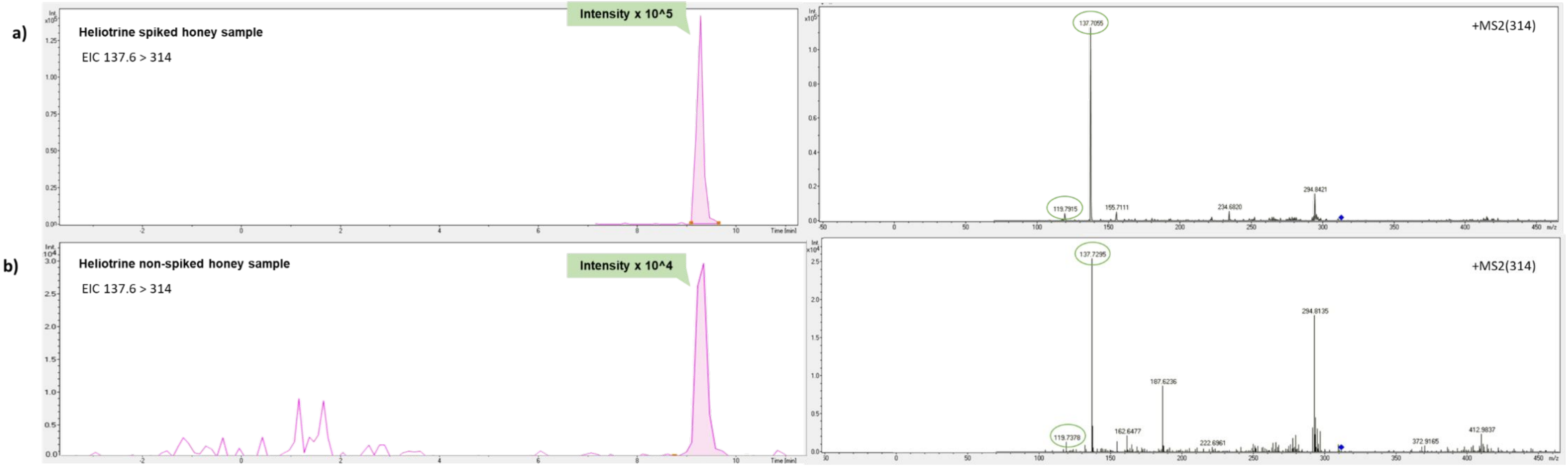

Figure S1. Extracted ion chromatograms and mass spectra (MS^2) of heliotrine in **(a)** honey sample spiked with a standard solution at a concentration of 25 µg/kg and **(b)** honey sample non-spiked and naturally contaminated with heliotrine.

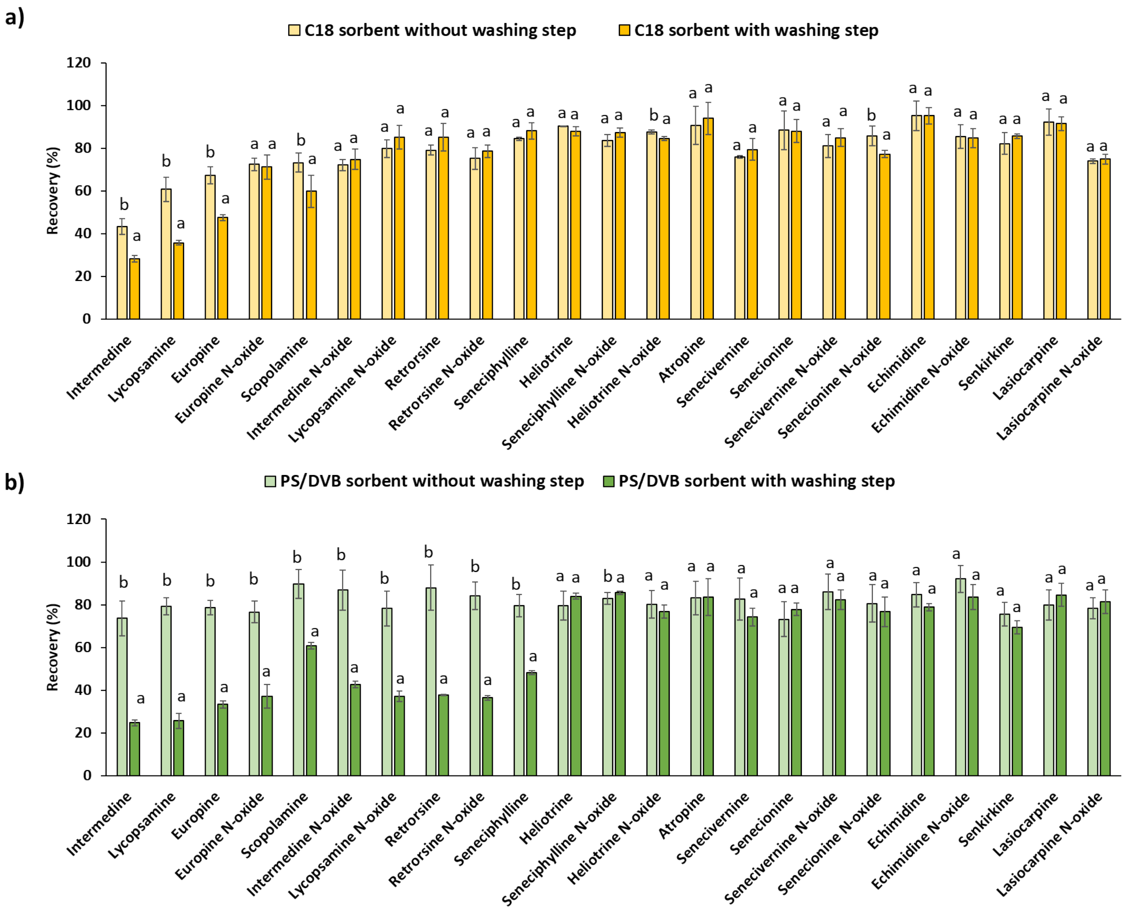

Figure S2. Recovery values obtained using (a) C18 and (b) PS/DVB µSPEed® cartridges from the analysis of honey samples spiked with the target analytes (50 µg/kg of each analyte) performing and not performing a washing step during the µSPEed® procedure before elution. Same letters means that there are no statistically significant differences ($p > 0.05$) and different letter means that there are significant differences ($p \leq 0.05$). Statistical analysis performed using Student T-test.

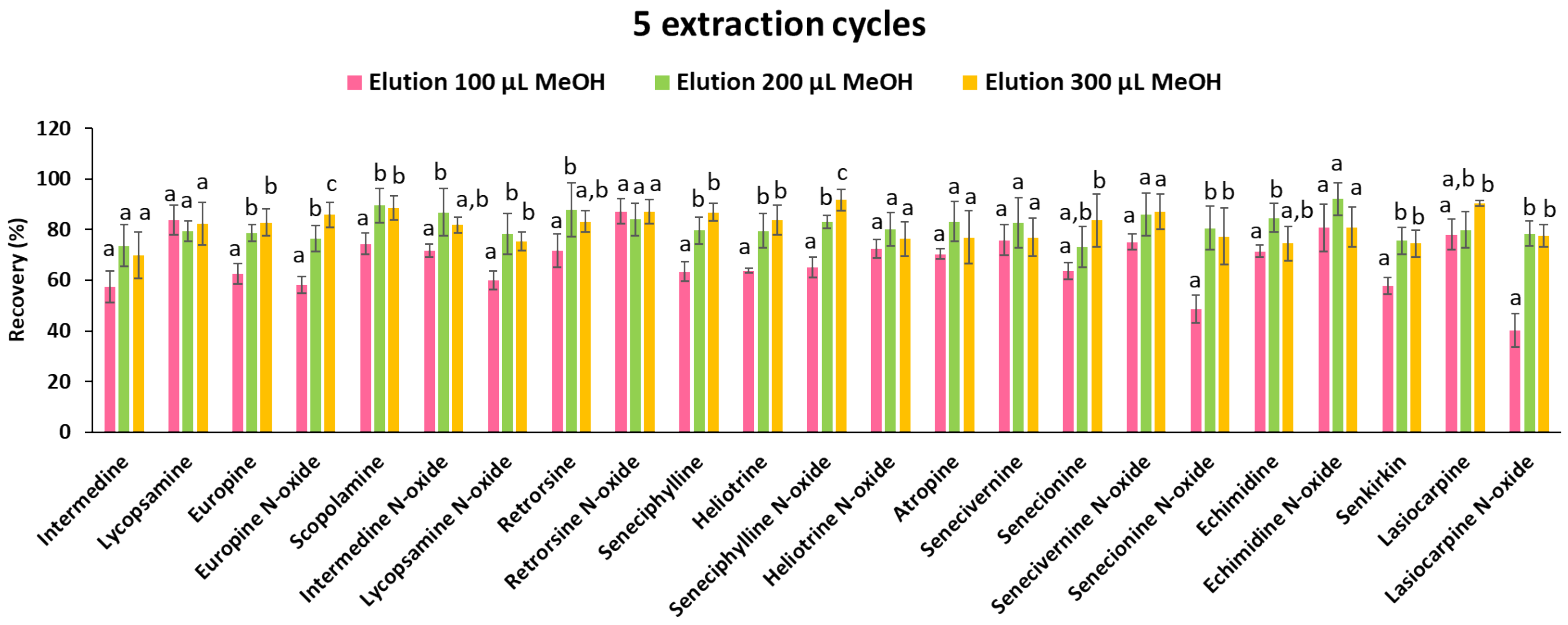

Figure S3. Recovery values obtained with PS/DVB µSPEed® cartridges from the analysis of honey samples spiked with the target analytes (50 µg/kg of each analyte) performing 5 extraction cycles and using different elution volumes of Methanol (MeOH). Same letters means that there are no statistically significant differences ($p > 0.05$) and different letter means that there are significant differences ($p \leq 0.05$). Statistical analysis performed using ANOVA and Duncan post-hoc multiple range test.

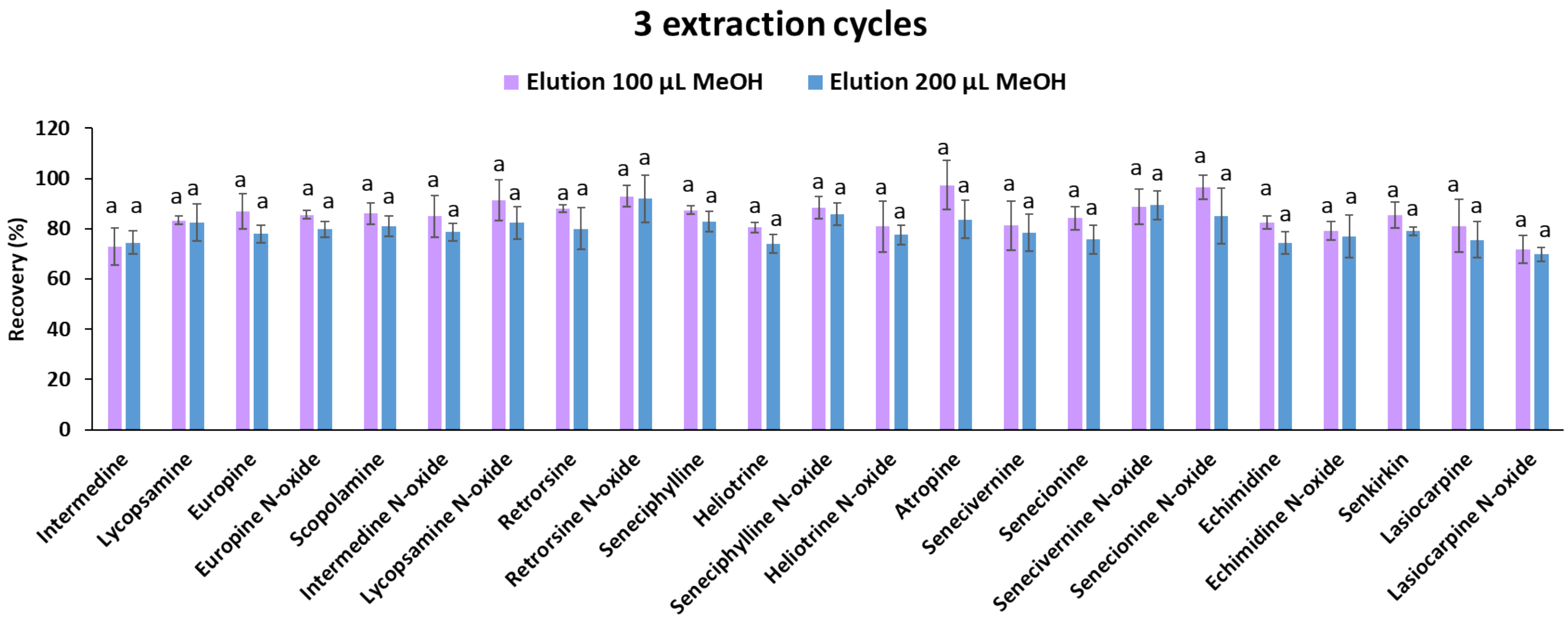

Figure S4. Recovery values obtained with PS/DVB µSPEed® cartridges from the analysis of honey samples spiked with the target analytes (50 µg/kg of each analyte) performing 3 extraction cycles and using different elution volumes of methanol (MeOH). Same letters means that there are no statistically significant differences ($p > 0.05$) and different letter means that there are significant differences ($p \leq 0.05$). Statistical analysis performed using Student T-test.

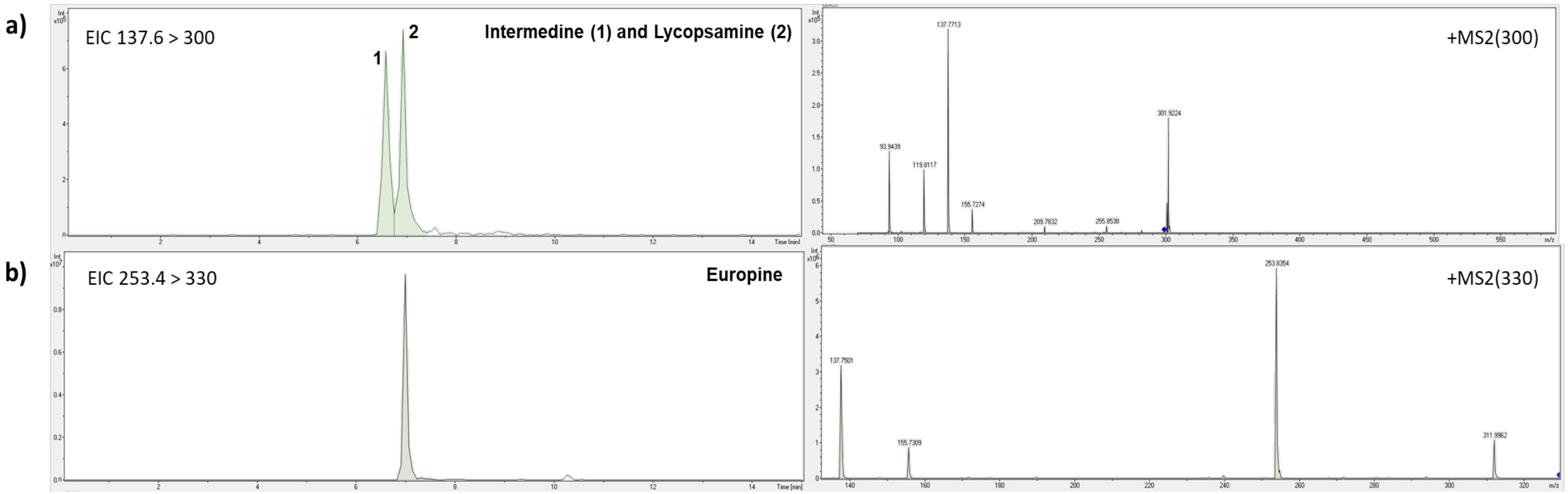

Figure S5. Extracted ion chromatograms and mass spectra (MS^2) of **(a)** intermedine and lycopsamine and **(b)** europine in a standard solution of 500 μg/L using the chromatographic method described in Section 2.4. MS^2 of intermedine and lycopsamine are the same because they are isomers, thus the MS^2 showed in the image corresponds to the one obtained from intermedine (peak 1).

Table S3. Input used to assign AGREEprep scores for µSPEed®-UHPLC-IT/MS/MS method.

Criterion	Input	Justification for input	Score	Weight
#1 (Sample preparation placement)	On site	Although in this work the sample preparation is performed in the lab, it could be possible to perform it on site because the digital device to perform µSPEed® can be brought to the sampling site if there is a power outlet.	0.33	1
#2 (Hazardous materials)	0.11	7.3 µL of H_2SO_4 and 100 µL of MeOH per sample.	0.65	5
#3 (Sustainability, renewability, and reusability of materials)	> 75% of reagents and materials are sustainable or renewable	Approximately 2.9 mL of water are considered renewable, what implies > 75% of the reagents used. On the other hand, although µSPEed® (4 mg) cartridges are not sustainable or renewable they can be used several times (up to 30-50 times).	0.75	2
#4 (Waste)	1.1	Sum of filter, 4 mg µSPEed® cartridge, 0.6 mL MeOH (elution and cleaning of cartridge), 0.2 mL of water because in contact with H_2SO_4, and 0.3 mL sample. However, the amount of sample and cartridge has been corrected dividing the amount of reusable materials by the number of times they can be used.	0.61	4
#5 (Size economy of the sample)	0.5	Amount of honey used.	0.77	2
#6 (Sample throughput)	15	15 min for sample dilution and homogenization, and 3 min per µSPEed® extraction, so 15 samples can be processed in 1 h.	0.64	3
#7 (Integration and automation)	3 steps – semi-automated system	Dilution, filtration and µSPEed® extraction. The µSPEed® extraction is a semi-automated system, the aspirate-dispense cycles are programed in the digital syringe	0.38	2
#8 (Energy consumption)	4.25 Wh per sample	Magnetic stirring (5 W) for 15 min, µSPEed® extraction with digital syringe (60 W) for 3 min	1.0	4
#9 (Post-sample preparation configuration for analysis)	Liquid chromatography (4th option)	UHPLC-IT-MS/MS is used for the analysis of the sample extracts.	0.25	2
#10 (Operator's safety)	3 hazards	3 pictograms in MeOH	0.25	3

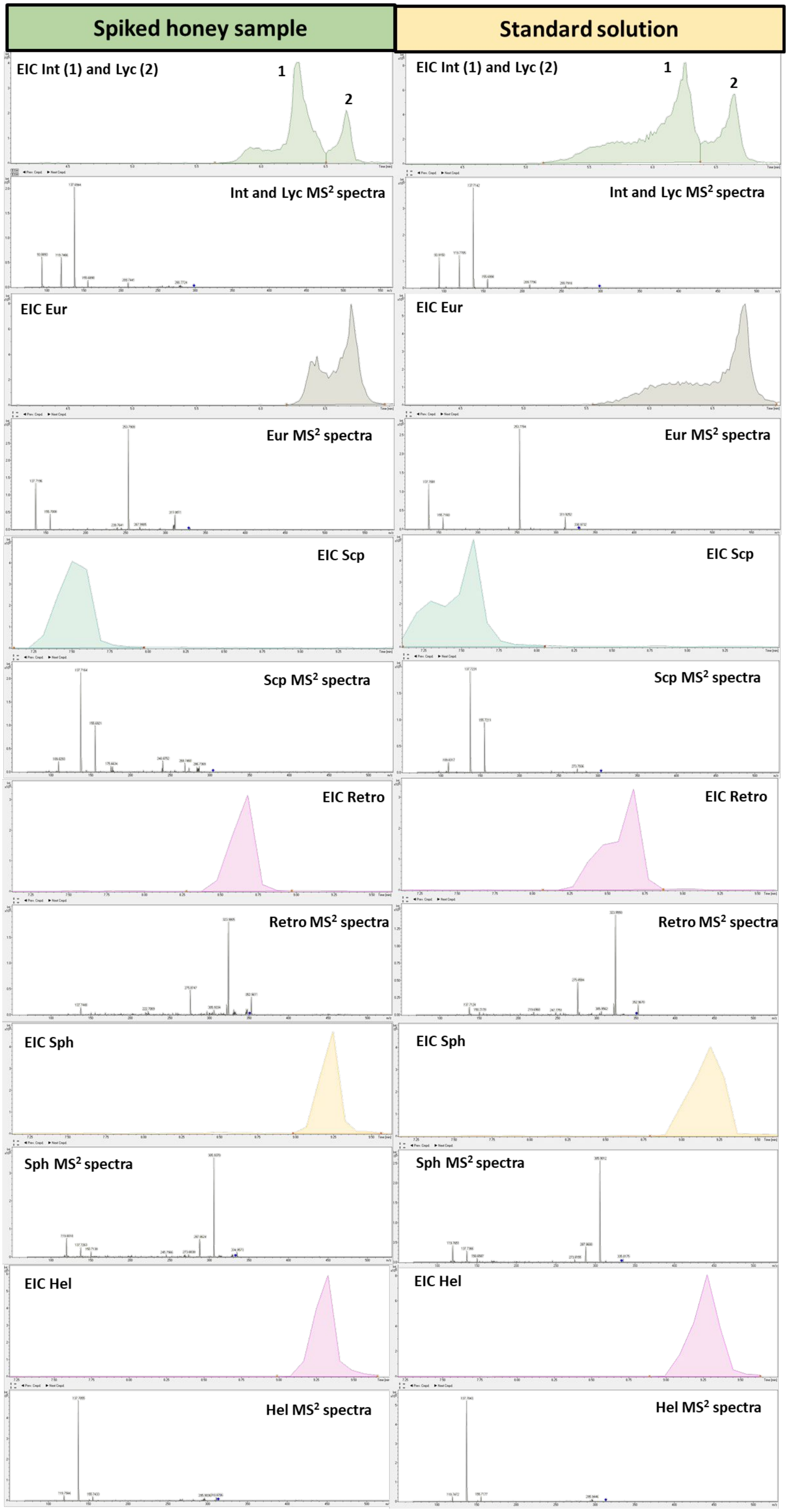

Spiked honey sample
Standard solution
EIC Int (1) and Lyc (2)
1
2
Int and Lyc MS^2 spectra
EIC Eur
Eur MS^2 spectra
EIC Scp
Scp MS^2 spectra
EIC Retro
Retro MS^2 spectra
EIC Sph
Sph MS^2 spectra
EIC Hel
Hel MS^2 spectra

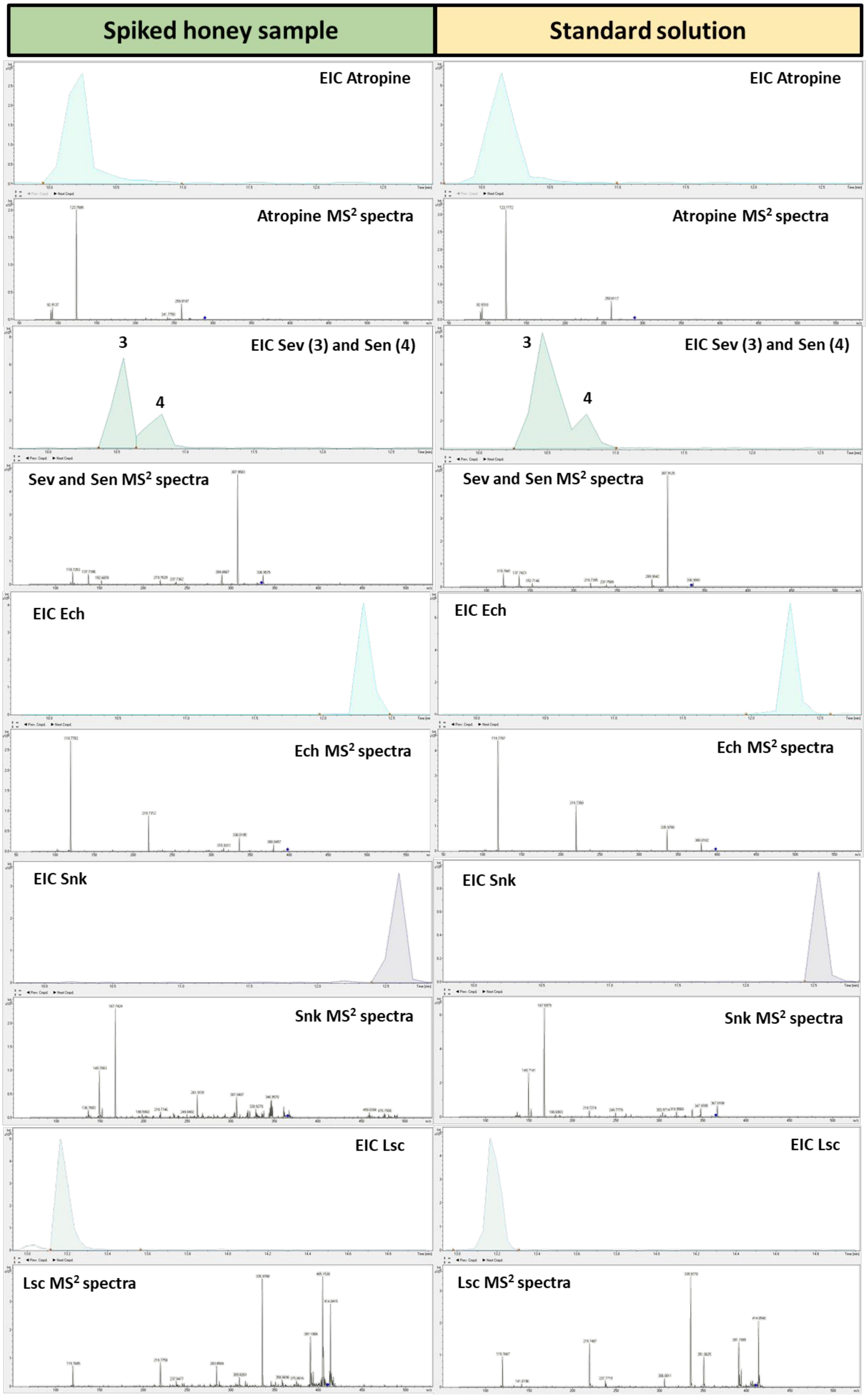

Figure S6. Extracted ion chromatograms (EIC) of product ions used for quantification and MS^2 spectra of pyrrolizidine and tropane alkaloids in spiked honey samples and standard solutions at the same concentration level (60 µg/L). Int: intermedine; Lyc: lycopsamine; Eur: europine; Scp: scopolamine; Retro: retrorsine; Sph: Seneciphylline; Hel: heliotrine; Sev: senecivernine; Sen: senecionine; Ech: echimidine; Snk: senkirkine; Lsc: lasiocarpine.

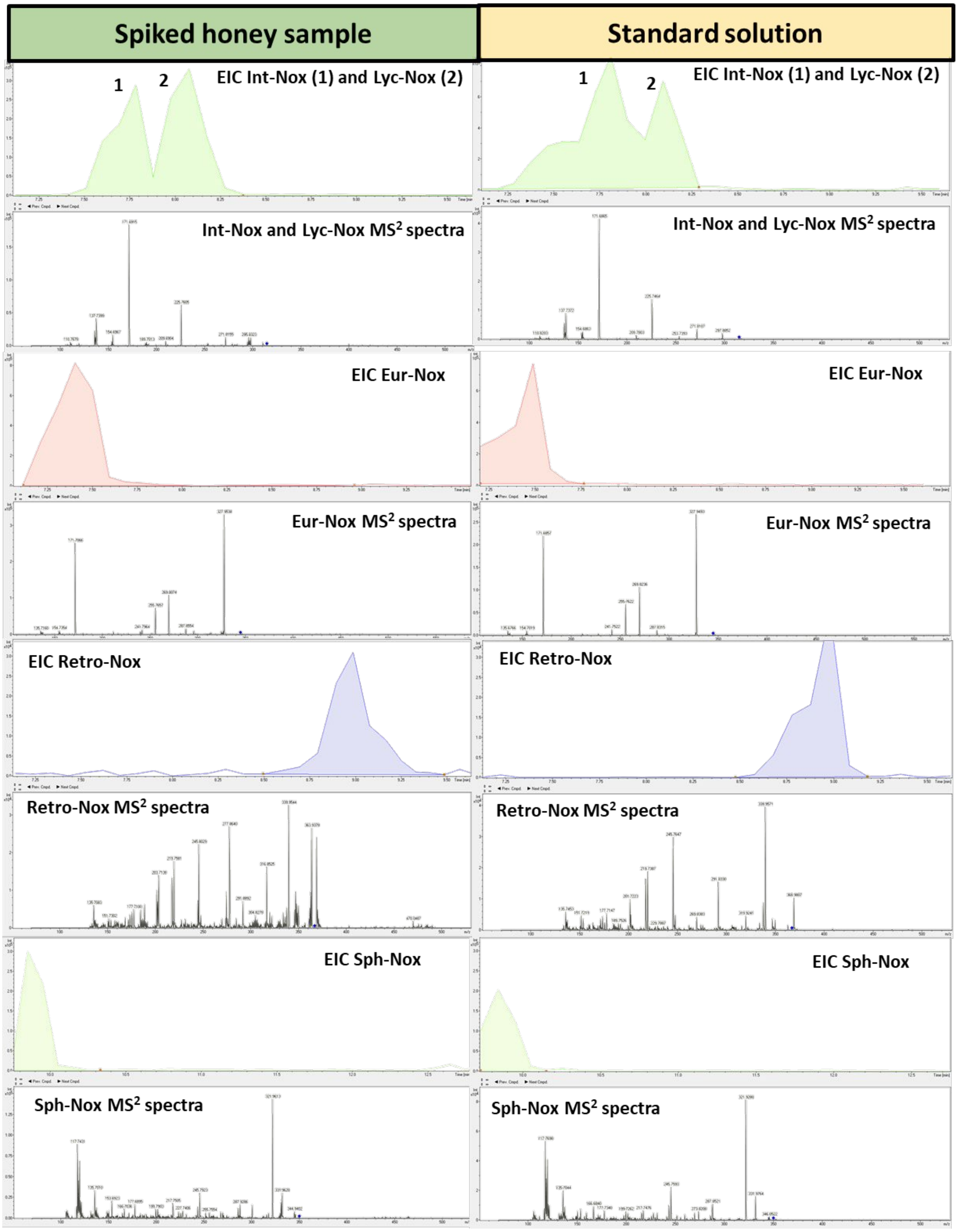
Spiked honey sample
Standard solution
EIC Int-Nox (1) and Lyc-Nox (2)
1
2
Int-Nox and Lyc-Nox MS2 spectra
EIC Eur-Nox
Eur-Nox MS2 spectra
EIC Retro-Nox
Retro-Nox MS2 spectra
EIC Sph-Nox
Sph-Nox MS2 spectra

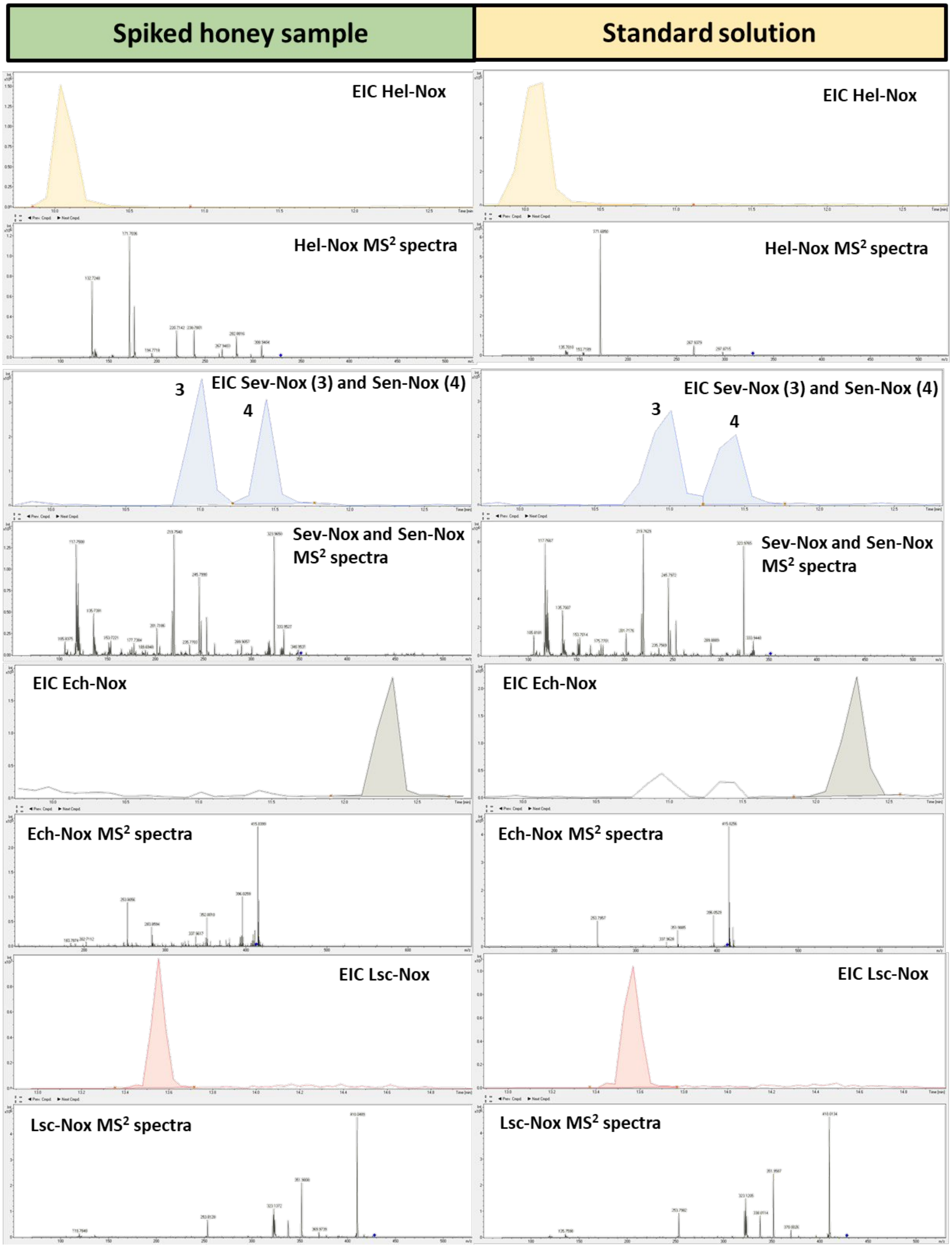

Figure S7. Extracted ion chromatograms (EIC) of product ions used for quantification and MS² spectra of pyrrolizidine *N*-oxides in spiked honey samples and standard solutions at the same concentration level (60 µg/L). Int-Nox: intermedine *N*-oxide; Lyc-Nox: lycopsamine *N*-oxide; Eur-Nox: europine *N*-oxide; Retro-Nox: retrorsine *N*-oxide; Sph-Nox: Seneciphylline *N*-oxide; Hel-Nox: heliotrine *N*-oxide; Sev-Nox: senecivernine *N*-oxide; Sen-Nox: senecionine *N*-oxide; Ech-Nox: echimidine *N*-oxide; Lsc-Nox: lasiocarpine *N*-oxide.

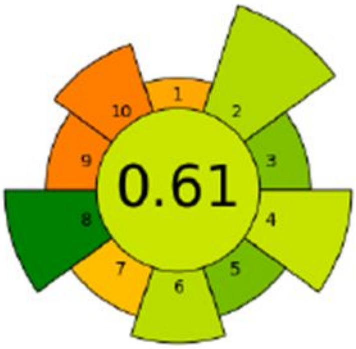

#	Criterion	Score	Weight
1.	**Sample preparation placement**<br>Sample preparation placement: On site	0.33	1
2.	**Hazardous materials**<br>Mass [g] or volume [mL] of problematic materials: 0.11	0.65	5
3.	**Sustainability, renewability, and reusability of materials**<br>> 75% of reagents and materials are sustainable or renewable	0.75	2
4.	**Waste**<br>Mass [g] or volume [mL] of waste: 1.1	0.61	4
5.	**Size economy of the sample**<br>Mass [g] or volume [mL] of the sample: 0.5	0.77	2
6.	**Sample throughput**<br>Hourly sample throughput: 15	0.64	3
7.	**Integration and automation**<br>No. of sample prep. steps: 3 steps; degree if automation: Semi-automated systems	0.38	2
8.	**Energy consumption**<br>Approximate energy consumption per analysis [W]: 4.25	1.0	4
9.	**Post-sample preparation configuration for analysis**<br>Liquid chromatography, gas chromatography with quadrupole detection, etc.	0.25	2
10.	**Operator's safety**<br>No. of distinct hazards: 3 hazards	0.25	3

Figure S8. Evaluation report of the AGREEprep assessment of the method proposed using µSPEed® for the determination of pyrrolizidine and tropane alkaloids in honey samples after applying the default weights of the tool

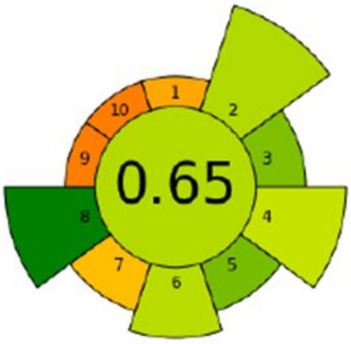

#	Criterion	Score	Weight
1.	**Sample preparation placement**<br>Sample preparation placement: On site	0.33	1
2.	**Hazardous materials**<br>Mass [g] or volume [mL] of problematic materials: 0.11	0.65	5
3.	**Sustainability, renewability, and reusability of materials**<br>> 75% of reagents and materials are sustainable or renewable	0.75	2
4.	**Waste**<br>Mass [g] or volume [mL] of waste: 1.1	0.61	4
5.	**Size economy of the sample**<br>Mass [g] or volume [mL] of the sample: 0.5	0.77	2
6.	**Sample throughput**<br>Hourly sample throughput: 15	0.64	3
7.	**Integration and automation**<br>No. of sample prep. steps: 3 steps; degree if automation: Semi-automated systems	0.38	2
8.	**Energy consumption**<br>Approximate energy consumption per analysis [W]: 4.25	1.0	4
9.	**Post-sample preparation configuration for analysis**<br>Liquid chromatography, gas chromatography with quadrupole detection, etc.	0.25	1
10.	**Operator's safety**<br>No. of distinct hazards: 3 hazards	0.25	1

Figure S9. Evaluation report of the AGREEprep assessment of the method proposed using µSPEed® for the determination of pyrrolizidine and tropane alkaloids in honey samples modifying the weight of criteria No. 9 and 10 in the tool to the lower value.

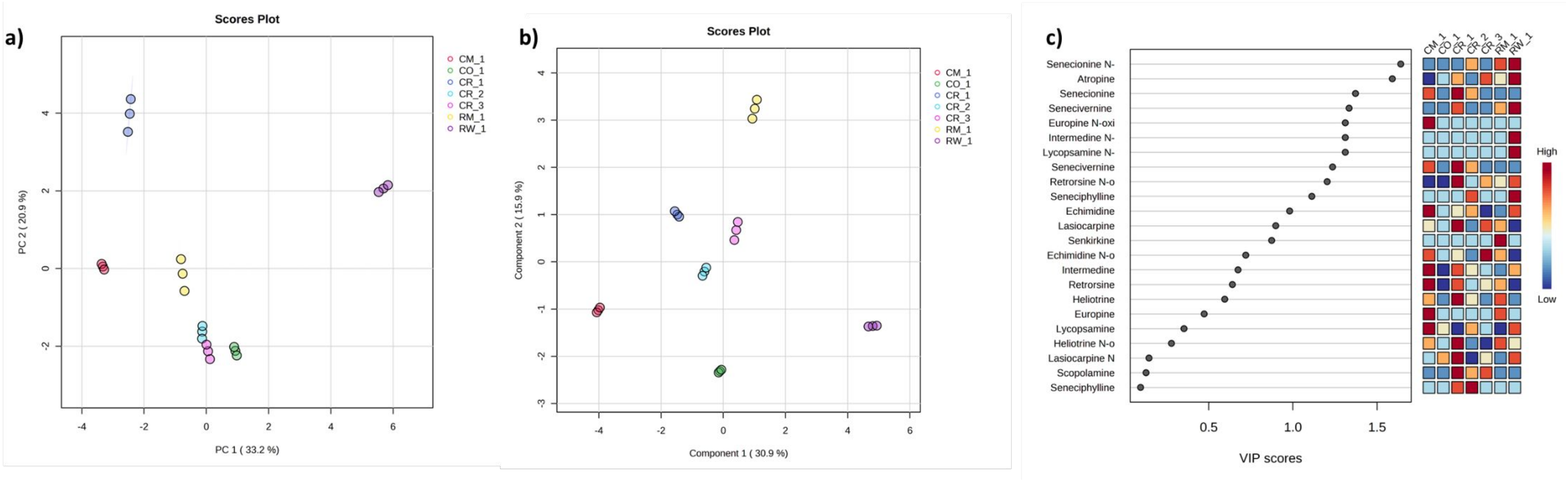

Figure S10. Multivariate statistical analysis (MVSA) using principal component analysis (PCA) and partial least square-discrimination analysis (PLS-DA) of the pyrrolizidine and tropane alkaloids determined in the honey samples analyzed. (a) PCA score plot, (b) PLS-DA score plot and (c) target alkaloids based on variable importance in the projection (VIP) score contributing to the variance observed in the PLS-DA model.
